# Finding the infectious dose for COVID-19 by applying an airborne-transmission model to superspreader events

**DOI:** 10.1371/journal.pone.0265816

**Published:** 2022-06-09

**Authors:** Mara Prentiss, Arthur Chu, Karl K. Berggren

**Affiliations:** 1 Department of Physics, Harvard University, Cambridge, MA, United States of America; 2 QVT Family Office, New York, NY, United States of America; 3 Department of Electrical Engineering and Computer Science, Massachusetts Institute of Technology, Cambridge, MA, United States of America; VIT University, INDIA

## Abstract

We probed the transmission of COVID-19 by applying an airborne transmission model to five well-documented case studies—a Washington state church choir, a Korean call center, a Korean exercise class, and two different Chinese bus trips. For all events the likely index patients were pre-symptomatic or mildly symptomatic, which is when infective patients are most likely to interact with large groups of people. Applying the model to those events yields results that suggest the following: (1) transmission was airborne; (2) superspreading events do not require an index patient with an unusually high viral load; (3) the viral loads for all of the index patients were of the same order of magnitude and consistent with experimentally measured values for patients at the onset of symptoms, even though viral loads across the population vary by a factor of >10^8^. In particular we used a Wells-Riley exposure model to calculate *q*, the total average number of infectious quanta inhaled by a person at the event. Given the *q* value for each event, the simple airborne transmission model was used to determined *S*_*q*_, the rate at which the index patient exhaled infectious quanta and *N*_0_, the characteristic number of COVID-19 virions needed to induce infection. Despite the uncertainties in the values of some parameters of the superspreading events, all five events yielded (*N*_0_∼300–2,000 virions), which is similar to published values for influenza. Finally, this work describes the conditions under which similar methods can provide actionable information on the transmission of other viruses.

## Introduction

After an initial period of controversy, it is now widely acknowledged that aerosols are a significant channel for transmission of COVID-19, and that separation by distances large enough to allow large droplets to settle ballistically (e.g. 2 meters) is not sufficient to reliably prevent transmission [1–4]. Many approaches can provide insight into whether or not virus transmission is dominantly airborne. One approach applies an aerosol transmission model to certain well-known cases, such as the Skagit Choir case from March 2020, where 32 out of 61 singers were confirmed positive after 2.5 hours of singing practice [5, 6].

Using such a model to probe whether viral transmission can be airborne requires the following steps: (1) apply the airborne model to known event parameters to determine the rate at which infectious quanta are emitted based on the measured infection rate for the event; and (2) test whether the rates extracted from different events are consistent. If the extracted rates are consistent for events with very different parameters, one can argue that transmission at those events was dominantly airborne. If the extracted values are not consistent, then applying the airborne model does not provide useful information about transmission mechanism, suggesting that aerosol transmission may not have played a role, that the model does not accurately describe the situation, or that the rate of quanta emission that drive infection is highly variable across individuals.

Superspreading events offer a useful template for probing airborne viral transmission, and comparing results from multiple events allows us to test the validity of the above assumptions, including the consistency of quanta emission rates. The large number of people involved in superspreading events also offers improved counting statistics in comparison with events involving only a few people. Furthermore, studying multiple superspreading events may provide insight into whether superspreading events are due primarily to an unusually high viral load in the index patient or can be observed given more conventional viral loads and the right environmental conditions (e.g. long exposures or activities with amplified aerosol emission). Miller *et al*. analyzed the Skagit Choir case in detail and estimated the quanta emission rate during the event at ∼1000 quanta/hour [6]. Similarly, using cases such as the Skagit case and the Diamond Princess, Bazant and Bush have published a detailed indoor guideline that can be used to estimate aerosol risks in varying activities [4]. Jimenez has published a similar calculator that estimates aerosol infection risks in various scenarios [7]. Both types of risk estimates are valid if one makes the following assumptions: (1) *S*_q_, the rate at which infectious quanta are emitted for a given activity, is similar across index patients; (2) the virus was distributed fairly uniformly over the volume of the venue; (3) the infection susceptibility to a given viral dose was similar for all attendees; and (4) the calculated emission quanta are not highly sensitive to small variations in input parameters, such as air exchange rates. For a new virus whose transmission has not yet been studied, it cannot be assumed that these assumptions are all valid.

In this work, we analyze the five superspreading events listed in the abstract, finding some results consistent with the existing literature but others which differ. In particular, we employ a standard physical model (well-mixed assumption and Wells-Riley susceptibility model) to calculate the rate of quanta emission ${\hat{S}}_{\text{q}}$ for index patient in each event [8]. This work and Bazant *et. al*. (who also assumed the virus was distributed uniformly over the volume of the venue) both estimate a single emission rate when only breathing or only talking (${\hat{S}}_{\text{q,breathing}}$ or ${\hat{S}}_{\text{q,talking}}$). These single-activity emission rates are fairly consistent with other published estimates [6, 7, 9]. More importantly, consistent with aerosol transmission as an infection mechanism and the first assumption above, these estimates are reasonably consistent across events with a broad range of input parameters. We note that the consistency of the ${\hat{S}}_{q}$ values for different events in this work and previous work is quite remarkable since measured viral loads for COVID-19 patients vary over 8–9 orders of magnitude [10–12]; we interpret the consistency of the quanta results in light of recent longitudinal studies by Goyal *et al*., which find a narrow time window (∼ 1 day) of peak viral loads that center around 10^7^ copies/mL [13, 14].

We also go on to calculate *N*_0_, the characteristic number of virions necessary to induce infection in the original SARS-CoV-2 strain, which requires estimating both the volume of aerosols emitted and the viral load (virions per unit volume) emitted from the index patient, neither of which were measured at the time of the five events studied. We thus estimate these values based on the literature. We find *N*_0_ values for the five cases ranging between 322 and 2,012 virions, with an average of ${\hat{N}}_{\text{0}}∼600$. Our range of *N*_0_ values is also consistent with recent work of Gale [15], who uses a very different method (thermodynamic response model) to estimate an ID_50_ for SARS-CoV-2 of ∼500 virions (*N*_0_ of 500/ln(2) ∼ 700 virions). We also note the similarity of this dose to the ∼300–9,000 copy inhaled infectious dose for Influenza A [16–18].

The estimated value and interpretation of *N*_0_ calculated here differ from other works [4, 6]. Bazant et al. calculate *N*_0_ of ∼ 10 virions, which the authors associate with a viral load of ∼ 10^9^ copies/mL [4]. Miller *et al*. assume *N*_0_ = 1000 virions, within the range found in this work, but infer a viral load of order 10^11^ copies/mL for the Skagit choir case [6]. These viral loads are in the 90th or higher percentiles of measured distributions [11]. If index patients did have such high viral loads, superspreading events would require superspreaders (exceptionally virulent individuals) and airborne transmission of COVID-19 would be rare.

In contrast, the *N*_0_ values in this work are associated with typical peak viral loads (∼ 10^7^ copies/mL) found in a short window around peak infectivity [13, 14]. The difference in *N*_0_ values and viral loads arises because Bazant *et al*. and Miller *et al*., assume that the volume of fluid emitted by an index patient per unit time is orders of magnitude smaller than our estimate. This difference is important if one tries to use transmission at superspreading events to calculate risks in other settings: if only patients with the highest viral loads emit quanta at the rate of the index patients in the superspreading events, then the risk of aerosol transmission would be very low at events attended by only a few people. In contrast, if, as this work suggests, patients with more typical viral loads drove the studied superspreading events, the airborne model predicts that there is a significant probability that the virus will be transmitted at small indoor gatherings with durations as small as an hour, consistent with observed results for COVID-19 transmission.

In the following, we first review the airborne transmission model, which is well-known in the literature. We then discuss some of the key model inputs and the wide range of measured values in the literature—particularly the volume of aerosols emitted per unit time, which directly governs the rate of virion emission. The Supporting Information contains a detailed discussion of the choices and ranges of input parameters used in this analysis and associated consistency checks. We also present a parametric analysis showing that varying the input parameters does not change the order of magnitude of *N*_0_.

## Materials and methods

We use two models: (1) a well-known “box” airborne virus transmission model; and (2) a similarly straight forward viral exhalation rate model. By comparing the predictions of these models to the recorded outcomes in the case studies we consider, and then applying a standard dose-response infection model, we are able to determine ${\bar{S}}_{\text{q}}$, the average rate of quanta exhalation for the index patient of each case study and *N*_0_, the threshold virion dose corresponding to a single quantum. For reference, Table 1 summarizes the main symbols, their meanings and units used in this paper.

**Table 1:** List of variables.

Variable	Meaning	Units	From
*B*	breathing rate	m^3^/hr	literature
*C*	concentration of virions in air	virions/m^3^	case analysis
*C*_*eq*_	equilibrium value of *C*	virions/m^3^	case analysis
*n*	number of virions in room	virions	case analysis
*n*_*eq*_	equilibrium value of *n*	virions	case analysis
*N*	dose of virions inhaled	virions	case analysis
*N*_0_	threshold dose, i.e. virions per quanta	virions	case analysis
*q*	dose of quanta inhaled	none	case analysis
*r*	attack rate	infections/person	case raw data
$\bar{S}$	virion emission rate (time and activity averaged)	virions/hr	case analysis
${\bar{S}}_{\text{q}}$	quanta emission rate (time and activity averaged)	quanta/hr	case analysis
${\hat{S}}_{\text{q,breathing}}$	${\hat{S}}_{\text{q}}$ in quiet breathing only	quanta/hr	case analysis
${\hat{S}}_{\text{q,talking}}$	${\hat{S}}_{\text{q}}$ while talking only	quanta/hr	case analysis
${\hat{S}}_{\text{q,singing}}$	${\hat{S}}_{\text{q}}$ while singing only	quanta/hr	case analysis
*T*	exposure time	hr	case raw data
*V*	room volume	m^3^	case raw data
λ	removal rate	1/hr	case analysis
λ_air_	air removal rate	1/hr	case analysis
λ_deactivation_	viral inactivation rate	1/hr	literature
λ_filter_	filtration rate	1/hr	case analysis
$\bar{\phi }$	average liquid emission rate	mL/hr	case analysis
*ϕ*_breathing_	aerosol emission rate while quiet	mL/hr	literature
*ϕ*_talking_	aerosol emission rate while talking	mL/hr	literature
*ϕ*_singing_	aerosol emission rate while singing	mL/hr	literature
*ρ*	viral density in aerosols	virions/mL	literature

There is a large body of reporting on “superspreading” events, which generically are taken to mean events where many individuals are infected by one or more index patients [25]. However, we have found a relatively small number of cases where not only the attack rate (the fraction of susceptible individuals that are infected at the event), but also, the physical parameters of the event (interaction time, room volume) are reported or estimable. In addition, as we discuss below, we focus on cases where we can approximate the room air as well-mixed and the virion density as uniform throughout the room. There are several cases, such as in airplanes [26–28], a meat packing plant [29], and a restaurant in China [30, 31], where the physical parameters and attack rate are well-characterized, but where the room has complex airflow and a clear spatial dependence of attack rates (e.g. air conditioning or other recirculating air patterns).

**Fig 1:**
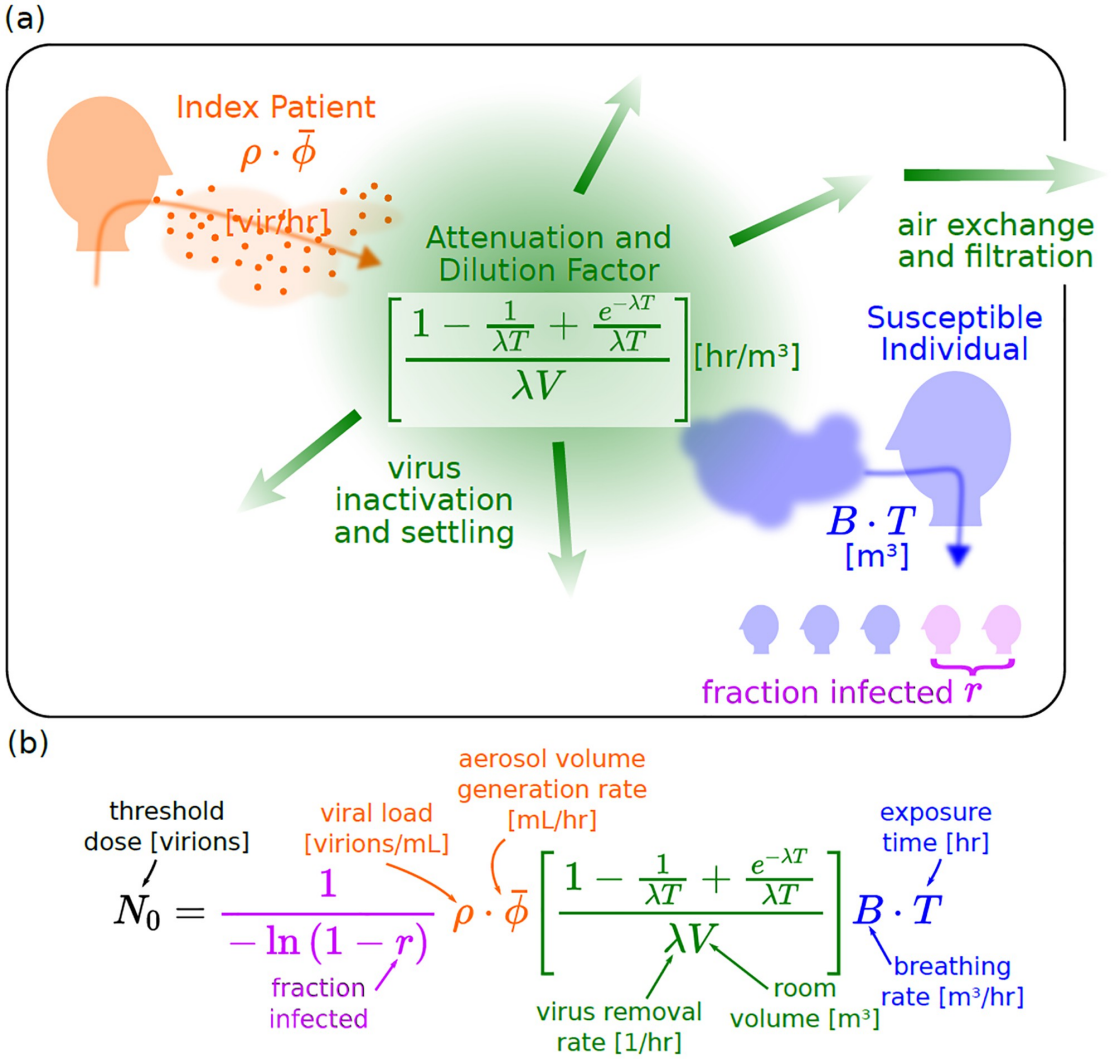
(a) A visual representation of the various terms used in part (a), illustrating their physical origin. (b) Equation describing how an estimate for the threshold dose for infection, *N*_0_, can be inferred from case data. The equation naturally divides into several components, one originating from medical data on virion emission rates for index patients, another that amounts to an effective room volume and incorporates environmental factors, a term that accounts for the volume inhaled by the patient, and one from epidemiological data on the attack rate.

**Fig 2:**
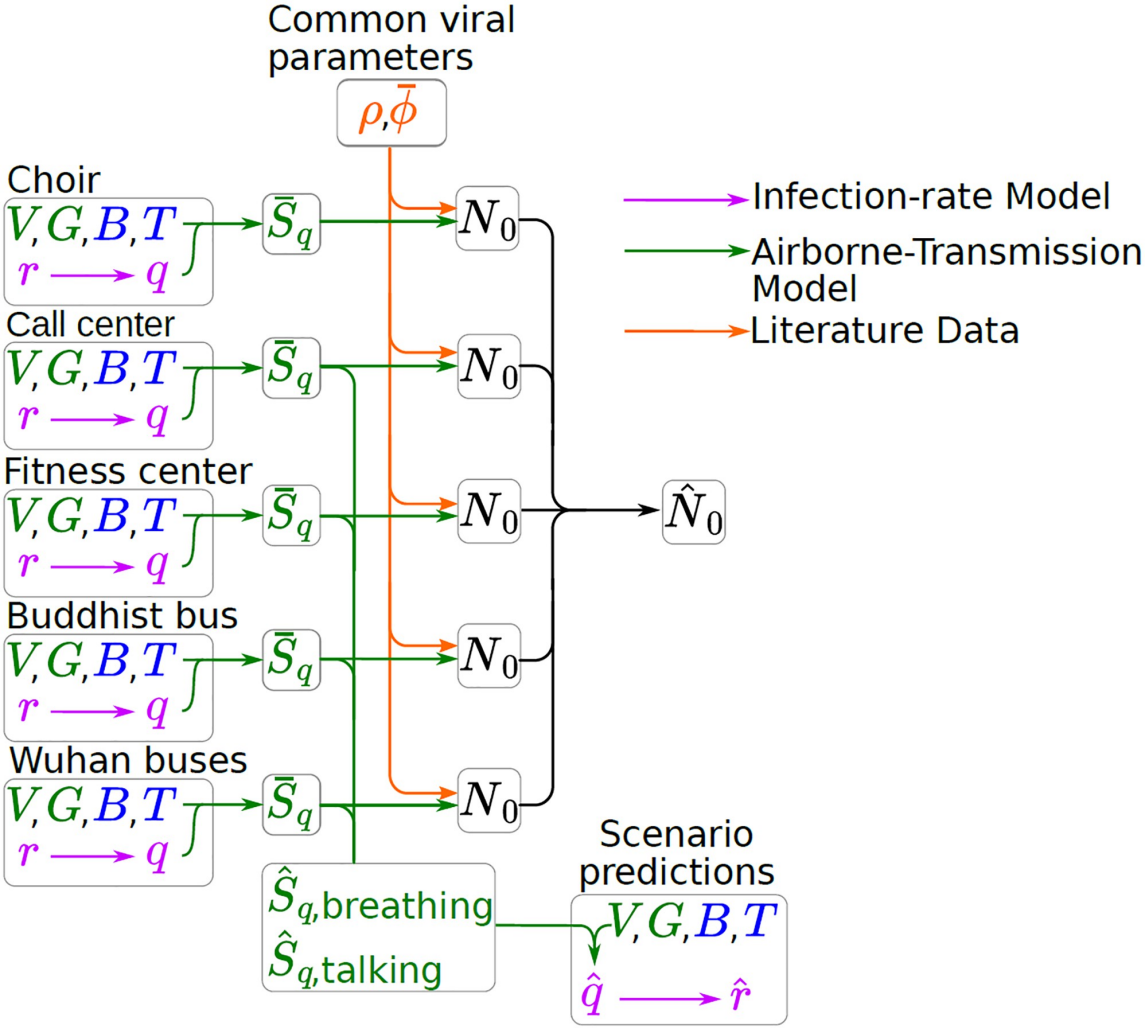
Flow chart illustrating the main conceptual thread of the paper, originating with raw data (either measured or inferred) from a series of case studies. The attack rate *r* from these data then fed into an infection-rate model (purple lines) which is used to estimate the inhaled quanta *q* for each case. A box airborne transmission model (green lines) is then used to calculate ${\bar{S}}_{q}$, the time-averaged rate of quanta emission from the index patient across the event. The mean of these values across cases is used to estimate ${\hat{S}}_{q}$ (for each activity). We can use this estimate (1) to predict $\hat{r}$, the attack rate of a hypothetical future event, and (2) to infer ${\hat{N}}_{0}$ by using literature values for infective load *ρ* and viral emission rate $\hat{\phi }$ combined with the estimated ${\hat{S}}_{q}$ above.

Figs 1 and 2 summarize the approach, which is discussed in detail in the Supporting Information. For the probability of infection after exposure to *N* infectious virions, we use a standard Wells-Riley model ($p(N)=1-e^{-N/N_{\text{0}}}$) [8]. In this version of the exposure model, calculation of the infectious virion exposure *N* requires knowledge of not only the physical parameters of the event (e.g. exposure time, room volume), but also, the rate of virion emission of the index patient (which in turn depend on the volume of aerosol production and the infectious viral load.) An equivalent formulation of the model is phrased in terms of quanta of exposure, where exposure to *q* quanta leads to a probability of infection *p*(*q*) = 1 − *e*^−*q*^. In this formulation, it is not necessary to know certain microscopic parameters (aerosol emission rate, viral load); rather, based on the observed attack rate (the fraction of people infected in a given case) and the physical parameters, the quanta emission rate *S*_q_ (having units of quanta per unit time) of the index patient is deduced. Furthermore, since both models give identical infection probabilities, 1 quantum of exposure is equal to an exposure of *N*_0_ virions. In the same way, the quanta emission rate *S*_q_ is equal to the virion emission rate *S* divided by *N*_0_, i.e. *S*_q_ = *S*/*N*_0_.

We applied the model to the following cases: (1) In January 2020, an index patient who rode a 100-minute round trip bus ride to a Buddhist worship event in China in January, infected 23 out of 67 bus riders. There was a second “control group” bus which went to the same event, in which 0 out of 60 riders were infected [32]. (2) Also in January 2020, an index patient in Hunan province, China took two different buses on a single trip. On the first 2.5-hour leg on a larger bus, 7 of 48 riders were infected, including riders ∼4.5 m away; on the second 1-hour leg on a smaller bus, 2 of 12 riders were infected [33]. (3) In February 2020, clusters of coronavirus transmission in aerobic dance exercise classes were found in Korea, this time due to sick instructors [34]. These classes were very short —only about 50 minutes—and 5–22 students were in a ∼60 m^2^, so that many were presumably separated from the instructor by more than 2 m. (4) In March 2020, 94 out of 216 employees working on the same floor of a large (∼1,000 m^2^) Korean call center tested positive. Of the positive results, 89 of them were on one side of the floor measuring over 400 m^2^ [35]. (5) Also in March 2020, 61 singers convened for 2.5 hours of choir practice in a ∼200 m^2^ church in Skagit County, Washington, in the U.S., after which 32 were confirmed positive and 20 were suspected positive [5, 6]. The index patients in these cases were believed to be mildly or pre-symptomatic, which is significant because the viral load (for the original SARS-CoV-2 strain) peaks around the time of symptom onset [13, 14].

**Fig 3:**
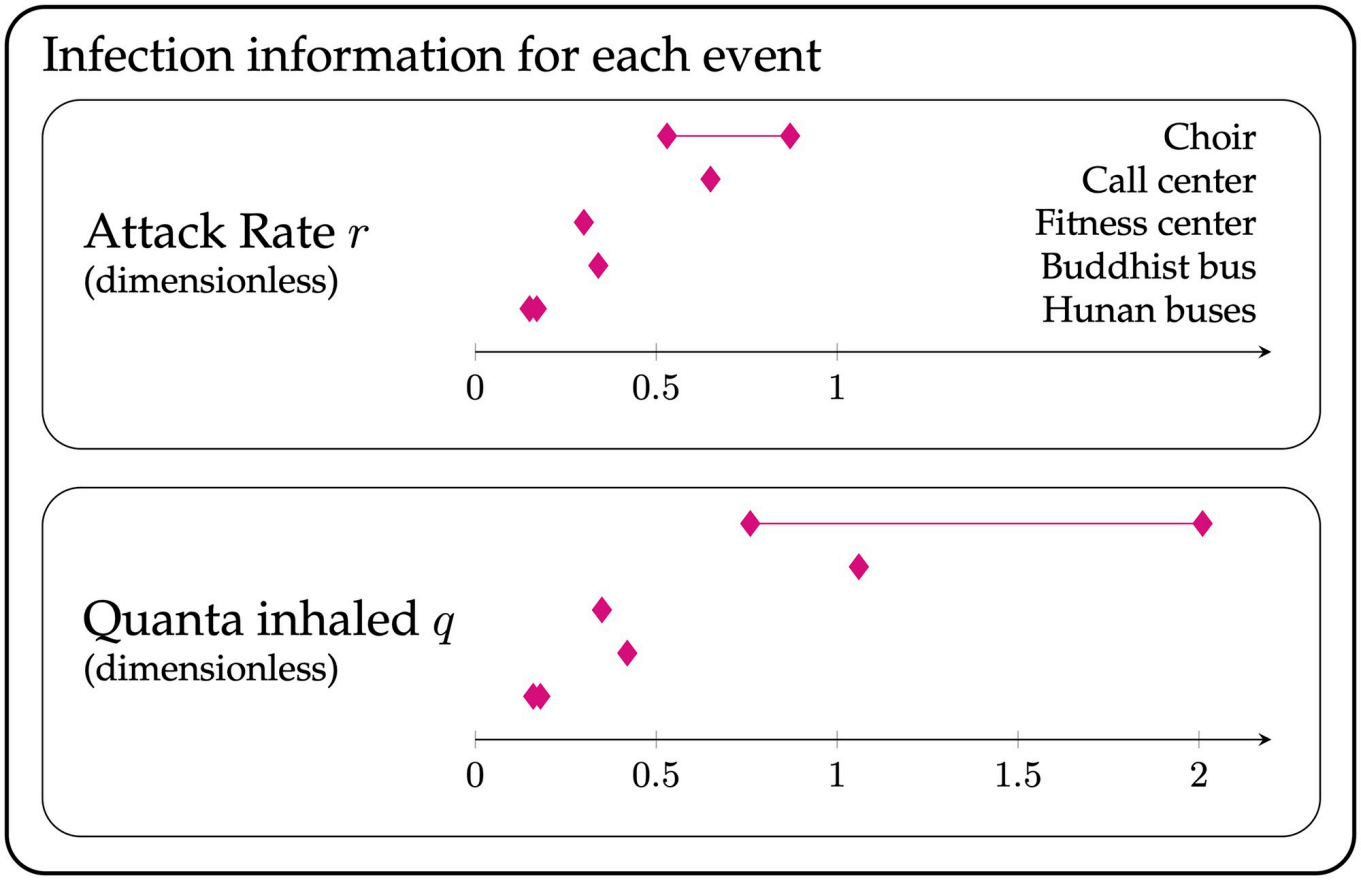
Attack rate *r* (infected / exposed) and quanta inhaled *q* = −ln(1 − *r*) for each of the five events. In the case of the choir, the smaller attack rate includes only confirmed cases, while the larger attack rate includes both confirmed and probable cases [5]. In the cases of the Hunan buses, the range spans the two bus rides.

**Fig 4:**
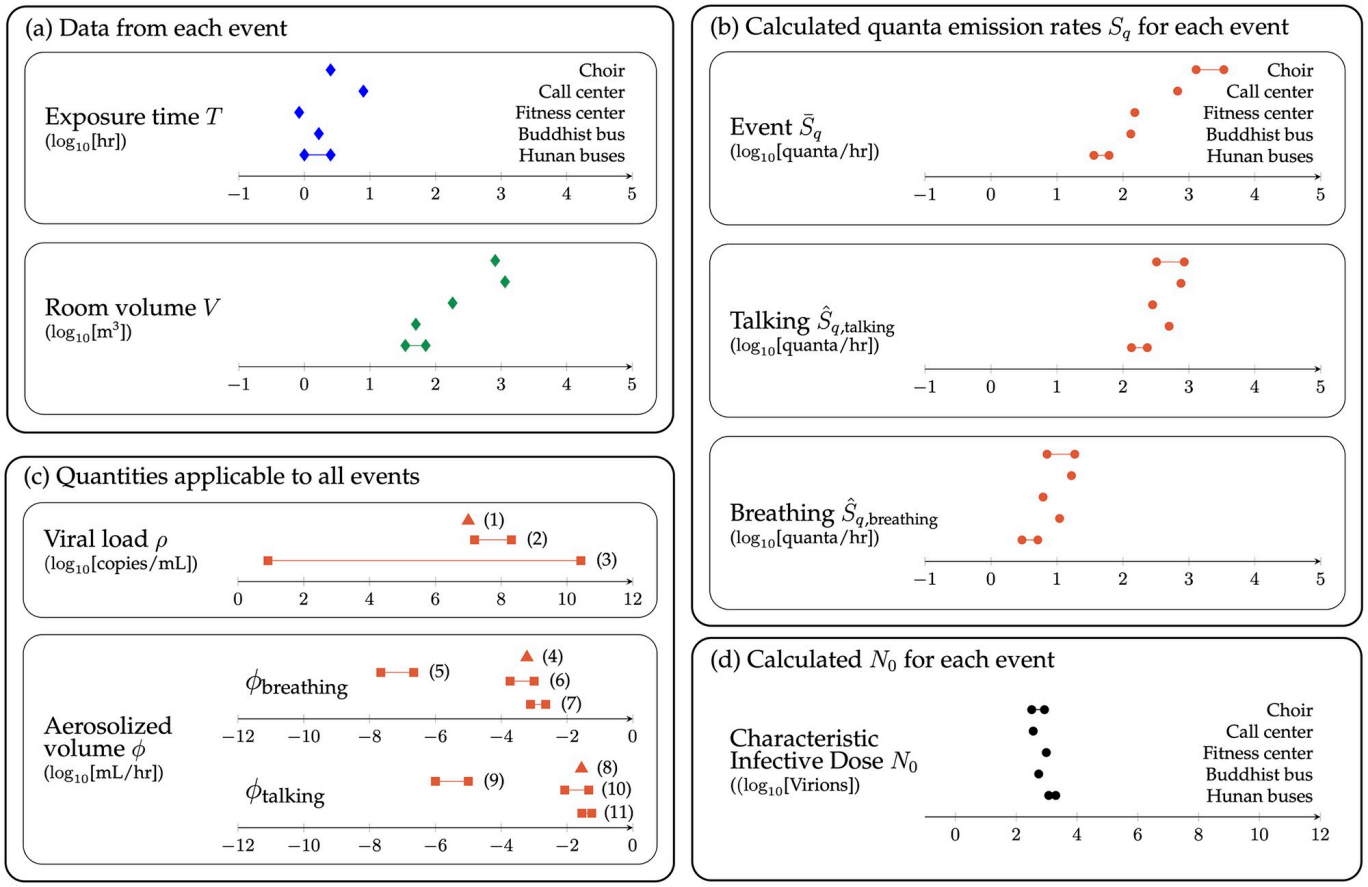
Key inputs and outputs. (a) Diamonds show the interaction time *T* and room volume *V* for each of the five cases, presented in the same order for both panels. Event 1 = Skagit choir, 2 = Korean call center, 3 = Korean fitness center, 4 = Buddhist bus, 5 = Hunan buses. (b) Estimated quanta emission rates for each case: *S*_*q*_ (event specific, top panel), *S*_*q*,talking_ (middle panel), *S*_*q*,breathing_ (bottom panel). Because each event has a different mix of activities (time spent singing/talking versus breathing), the estimated event quanta emission rate *S*_*q*_ ranges over 2 orders of magnitude. However, the estimated quanta emission rates for talking and breathing alone (*S*_*q*,talking_) range over only a factor of ∼6, which is also narrower than the range in *T* and *V*. (c) Values of viral load *ρ* and aerosolized volume *ϕ* used in this work (triangles) and in literature (circles). *ρ*: (1) value used in this work (10^7^ copies/mL); (2) modeled peak viral load found by Goyal *et al*. across 25 patients [13]; (3) range observed by Kleiboeker *et al*. across 4,432 patients [11]; *ϕ*: Breathing (4) value used in this work (6.0 × 10^−4^ mL/hr); (5) range implied from Miller *et al*. based on Morawska *et al*. [6, 19]; (6) range implied from Stadnytskyi *et al*. (using ratio of breathing/speaking in Morawska) *et al*. [19, 20]; (7) range based on exhaled breath condensate (EBC) dilution measurements [21–23]; Talking (8) value used in this work (2.7 × 10^−2^ mL/hr); (9) range in Miller *et al*. and Morawska *et al*. (1–10 nL/hr) [6, 19]; (10) range in Stadnytskyi *et al*. [20]; (11) range in Smith *et al*. [24]; (d) Estimated characteristic infective dose *N*_0_ for each of the five cases.

Each of the five cases provides an infection rate (also called attack rate) *r* equal to the fraction of exposed individuals who were infected. *r* can be used to then estimate *q* in each case (Fig 3). We can also calculate *q* based on a standard “box” model of airflow (also called a well-mixed model), as a function of the various parameters that characterize the event [6, 9, 36]. Fig 1 illustrates the model principle and key parameters: an index patient releases a volume of aerosols per unit time at $\bar{\phi }$, and each aerosol contains *ρ* virions/unit volume. Equivalently, the index patient releases infectious quanta at a rate ${\bar{S}}_{q}$, where, by definition of a quanta, $\rho \cdot \bar{\phi }={\bar{S}}_{q}N_{\text{0}}$. Air exchange and other factors cause infectious virus to decay at a rate λ. The overall concentration of virions is governed by a dimensionless dilution factor *G*, which is a function of the virion decay rate λ, the room volume *V*, and an interaction time *T*. Susceptible individuals breathe in virus-laden air at a rate *B*, resulting in a fraction *r* of them becoming infected. Fig 2 shows how each case’s physical or epidemiological parameters (*V*, *G*, *B*, *T*, *r*) give a value of ${\bar{S}}_{q}$ for that case, which are averaged across cases to give an estimate of the emission rate ${\hat{S}}_{q}$ used to estimate the infection rate $\hat{r}$ in a hypothetical scenario. In each case, the observed ${\hat{S}}_{q}$ results from different activities (e.g. singing in the choir case, versus largely breathing in the bus ride cases); we use relationships between relative expulsion rates in different activities from the literature to standardize the quanta emission rates for breathing ${\hat{S}}_{q,\text{breathing}}$ or talking alone ${\hat{S}}_{q,\text{talking}}$ (Fig 4).

The breathing rate *B*, the decay rate λ, and the source term $\bar{S}$ are estimated based on a combination of values from the literature and analysis of case-specific data. The source term $\bar{S}$, which has units of virions/hour, can be calculated as a product of the volume of aerosols per unit time released by the index patient ($\bar{\phi }$) and the density of virions in those aerosols (*ρ*) (Fig 1); both parameters are based on values from the literature. We refer the reader to the Supporting Information for a more detailed discussion.

### Viral density *ρ*

This quantity has been directly measured, but it ranges over many orders of magnitude across patients (order 10^2^ copies/mL to 10^10^ copies/mL) [10–12, 37]. In addition, RT-PCR is sensitive to nucleic acid from infectious virions and non-infectious nucleic acid fragments. Furthermore, the infectious fraction of PCR-measured nucleic acid can change over time; however, near the onset of symptoms and at the peak in viral loads the infectious virions are of order of the total virions [38–42]. Thus, since we are considering patients near the onset of symptoms, we can assume that the viral loads measured by RT-PCR dominantly represent infectious virions. Furthermore, there is generally a narrow time period of maximum infectiousness (often coinciding with symptom onset), estimated by Goyal *et al*. to be approximately 0.5–1.0 day around the time of peak viral load [14]. Based on the viral trajectory of 16 hospitalized patients, Goyal *et al*. estimated a peak viral load, as measured by nasopharyngeal (NP) swab, of ∼7.2–7.3 Log_10_ copies/mL. We therefore use a value of *ρ* = 10^7^ copies/mL, which approximates the average viral density in the window of ∼1 day around the peak as modeled by Goyal *et al*. This is also the value that was used in Monte Carlo modeling of the individual infection risk for susceptible subjects confined in a room with an asymptomatic index patient [36]. As a side note, this value is also not far from the mean density measured by Wölfel *et al*., who found a mean viral density in sputum of 7.00 × 10^6^viral copies per mL (6.85 Log_10_ copies/mL) [40]. The assumed value of *ρ* is the ∼ 70th percentile of values measured by Kleiboeker *et al*. [11].

### Volume expelled per unit time *ϕ*

The value of *ϕ* (volume of aerosols expelled per unit time) depends on what the person is doing. We use measured values for talking and then infer the results for other activities such as talking, breathing, and singing [19, 43]. We also note that some individuals have been measured to be “superemitters”, releasing approximately an order of magnitude more fluids than typical persons [24, 43]; values of *ϕ* here are intended to represent typical emission volumes. Again, the literature includes a wide range of values, as we discuss in detail in the Supporting Information. In particular, Bazant *et al*. and Miller *et al*. [4, 6] use the measurements of Morawska [19], which show emitted volumes approximately three orders of magnitude lower than those measured by Stadnytskyi (and separately by Smith), which we use in this work [20, 24]. In the Supporting Information, we show that, after adjusting for the relative difference between volumes emitted when speaking versus breathing, the volumes in Stadnytskyi’s measurements are consistent with those in exhaled breath condensate dilution measurements [21–23]. In addition, we show that, when input into a simple well-mixed model, the volumes measured by Stadnytskyi accurately predict the measured volume of SARS-CoV-2 virions observed in a Singapore hospital ward [44]. We also discuss the differences in our calculation and the measurement of Ma *et al*., who find a much larger virion emission rate; we attribute this difference to the particular conversion of cycle threshold (Ct) value to viral loads used in Ma *et al*.’s work.

In sum, using the expression $\bar{S}=\rho \bar{\phi }$ for various activities: (1) *S*_breathing_ ∼ 6.0 × 10^3^ virions/hour; (2) *S*_talking_ ∼ 2.7 × 10^5^ virions/hour; and (3) *S*_singing_ ∼ 1.6 × 10^6^ virions/hour.

## Results

As noted previously, in each of the five case studies, an attack rate or range of attack rates (number of infected/ number of exposed) *r* is reported along with a description of the environment. From this information, along with the source and decay parameters discussed in the previous section, *N* and therefore *N* *_0_*, can be estimated from *r* = 1 − exp (*-N/N_0_*).

In estimating *N*, the physical parameters of the case studies are not all known with precision, and it is necessary to make assumptions about many of them. However, as we show in the parametric analysis below, the results are not greatly affected by varying the inputs within the ranges found in the literature. The Supporting Information details the assumed base case breathing rate, air exchange/decay rates, and virion expulsion rates. In addition, given the large difference in virion expulsion for different activities, we must make an assumption about the percentage of time spent talking/singing versus breathing. For choir singing, based on published observations, we assume that $\frac{2}{3}$ of the time is spent singing [45]; in the cases of the call center, fitness class, and buses, respectively, we assume that 90%, 50%, and 25% of the time are spent talking. Finally, there can be ambiguity in the attack rate: in the case of the Skagit Choir, there were 32 confirmed infected and 20 probable infected cases, so the attack rate ranges from 53–87%; we calculate *N*_0_ in both cases [5]. In the case of the Korean Fitness center, the reported attack rate across all events is 26% (57 confirmed/217 exposed); however, this includes an instructor teaching yoga and pilates (0 confirmed/25 exposed), which we exclude from the analysis [34]. Finally, in the case of the Korean call center, out of 94 total cases on the 11th floor, 89 of them were in an area separated from the rest of the floor by elevator banks and other rooms [35]; we calculate the attack rate for this area, but show that the value of *N*_0_ is similar if one instead uses the entire area.

Table 2 shows the key inputs and outputs for the calculations. For the interested reader, we include in the Supporting Information a spreadsheet where these inputs can be customized. For the base inputs, the range of *N*_0_ (322–2,012) that results from the five scenarios is reasonably narrow. The order of magnitude of *N*_0_ calculated is consistent with initial expectations of order a few hundred to a few thousand particles for SARS-CoV-2 [46]. In addition, under the assumption of an activity-independent emission rate of 3.3 × 10^4^ virions/hr (compared to our estimates of 6.0 × 10^3^ and 2.7 × 10^5^ virions/hr for breathing and speaking, respectively), Kolinski and Schneider estimate the number of inhaled virions (*N* in our notation) for various superspreader events. Although the value of *N* and *N*_0_ are logically distinct quantities, their finding of *N* in the range of 50–100 virions is broadly compatible with the value of *N*_0_ found in this work [47]. Finally, Gale estimates *N*_0_ using a completely different, thermodynamic equilibrium method: he finds an ID50 (virion exposure with a 50% probability of infection) of ∼ 500 virions, which corresponds, under a Wells-Riley model, to *N*_0_ of 500/ln(2) ∼ 720 virions. This result also accords with the range found in this work.

**Table 2:** Key inputs and outputs for the case studies. (Refer to the spreadsheet S1 Table in the Supporting Information for details). The quanta/hour (scenario average) refers to the quanta emission rate averaging the high and low attack rate cases, for the particular parameters of that scenario. The quanta/hour (talking, breathing) are the implied quanta/hour for talking and breathing.

	Skagit Choir	Korean call center	Korean fitness center	Buddhist Bus	Hunan (Bus 1)	Hunan (Bus 2)
**Inputs**						
Relevant activity	Singing	Talking	Talking	Talking	Talking	Talking
% of time in activity	67%	90%	50%	25%	25%	25%
% of time breathing quietly	33%	10%	50%	75%	75%	75%
Source term *S* (virions/hr)	1.1 × 10^6^	2.5 × 10^5^	1.5 × 10^5^	7.2 × 10^4^	7.2 × 10^4^	7.2 × 10^4^
λ_air_ (air exchange rate hr^−1^)	1.5	1.5	1.5	3.0	3.0	3.0
λ_deactivation_ (settling + inactivation hr^−1^)	0.6	0.6	0.6	0.6	0.6	0.6
Total decay rate λ (hr^−1^)	2.1	2.1	2.1	3.6	3.6	3.6
Total time *T* (hr)	2.5	8.0	0.8	1.7	2.5	1.0
Breathing rate *B* (m^3^/hr)	0.5	0.5	2.0	0.5	0.5	0.5
Volume of Space *V* (m^3^)	810	1,143	180	50	71	34
Attack rate *r* (infected /population)	53%-87%	65%	30%	34%	15%	17%
**Outputs**						
Viral copies/m^3^ (time averaged)	519	96	207	138	254	428
Viral copies inhaled *N*	649	384	345	230	317	214
***N*_0_ (viral copies)**	**322–851**	**361**	**980**	**547**	**2,012**	**1,175**
${\bar{S}}_{\text{q}}$ (quanta/hr, scenario average)	2,347	683	152	133	36	62
${\hat{S}}_{\text{q,talking}}$ (quanta/hr)	586	757	279	500	136	233
${\hat{S}}_{\text{q,breathing}}$ (quanta/hr)	13	17	6	11	3	5

We also calculate the quanta/hour emitted in each scenario (the number of *N*_0_ emitted by the index patient in each of the case studies), and the implied quanta/hr emitted by speaking (range 136–757, average of 461) and breathing (range 3–17, average of 10). To connect these values with *N*_0_, at the assumed viral load of 10^7^ copies/mL, talking releases 2.7 × 10^5^ virions/hour, and breathing releases 6.0 × 10^3^ virions/hour; thus the average quanta correspond to a value across scenarios of ${\hat{N}}_{0}$ of ∼ 600 viral copies. (As for ${\hat{S}}_{\text{q}}$, the ^ notation here over *N*_0_ indicates an average across scenarios).

These quanta are broadly consistent with those found by other authors: Miller finds a mean emission rate of 970 quanta/hr for the Skagit choir case [6]; Bazant finds ∼4–16 quanta/hr for breathing and 54–970 quanta/hr for talking and singing [4]. Similarly, although Evans uses smaller values for emitted aerosol volumes, these are offset by higher assumed viral loads, such that the net results of 60 quanta/hr for breathing and 600 quanta/hr for speaking are similar to those found here and by others [48]. Jimenez, based on the work of Buonanno *et al*., recommends a range of ∼2–14 quanta/hr for oral breathing (depending on the activity) and 61–408 quanta/hr for loud speaking [7, 9].

### Parametric analysis

A number of the inputs to the calculation were not directly measured (e.g. air exchange rate, breathing rate, percentage of time talking), or have a wide range in the literature (e.g. deactivation rates due to settling and viral inactivation). Here we perform Monte Carlo simulations that vary these parameters over representative ranges from the literature and calculate the resulting ranges of *S*_q_ and *N*_0_. While a different choice of ranges or distributions is possible, the results are not highly sensitive to these choices. The variables and their ranges are as follows:

1. Air exchange rate λ_air_: we vary λ_air_ by a range of ± 67%, so that in the office, choir, and fitness center cases a range of 0.5–2.5 hr^−1^, and in the bus case, a range of 1.0–5.0 hr^−1^, are considered. The EPA exposure factors handbook recommends a base value of 1.5 air exchanges/hour for nonresidential structures, with a measured standard deviation of 0.87 ACH/hour (∼ 60% of the base value) [49]. Similarly, the measured air exchange rates for automobiles in recirculate mode ranged in Tong *et al*. from 2.5–5.5 ACH, compared to our assumed value of 3.0 ACH and range of 1.0–5.0 ACH for the bus cases [50]. We also note that most cases considered here are from Asia, while the EPA recommendations are based on U.S. structures. The ranges considered here should be sufficiently broad to accommodate differences in standards across countries. For example, You *et al*. measured air exchange rates across various school classrooms, meeting rooms, and reading rooms in China and found average exchange rates of 0.73–1.91 hr^−1^ [51], within the 0.5–2.5 hr^−1^ range above. Similarly, based on a survey of measured values in Hong Kong, Gao *et al*. simulate airborne transmission in a city using air exchange rates of 1.0 for offices, shops, and restaurants, 1.4 for other public locations, 2.0 for classrooms, and 4.0 for transportation [52]. These values are again within the ranges used in the parametric study.
2. Viral attenuation and settling rate λ_deactivation_: we vary λ_deactivation_ by a range of ± 100% around the base value of 0.6 hr^−1^(range of 0–1.2 hr^−1^). For settling, Diapouli measures a settling range of ∼ 0.05–0.65hr^−1^ for PM 2.5–10 particles [53]; for viral deactivation, as discussed above, Van Doremalen and Fears, respectively, measure λ ∼ 0.6 and λ ∼ 0 hr^−1^ [54, 55].
3. Breathing rate *B*: we vary *B* by a range of ± 33%. Binnazi measures a standard deviation of breathing rates of 40–65% of the mean rate when quietly breathing, speaking, or singing; Adams measures standard deviations of 25% of the mean when sitting or standing [56, 57].
4. Percentage of time talking or singing: we vary this by a range of ± 20% of the base value in each scenario; for instance, in the fitness center case, the time spent talking varies from 40–60%.

**Fig 5:**
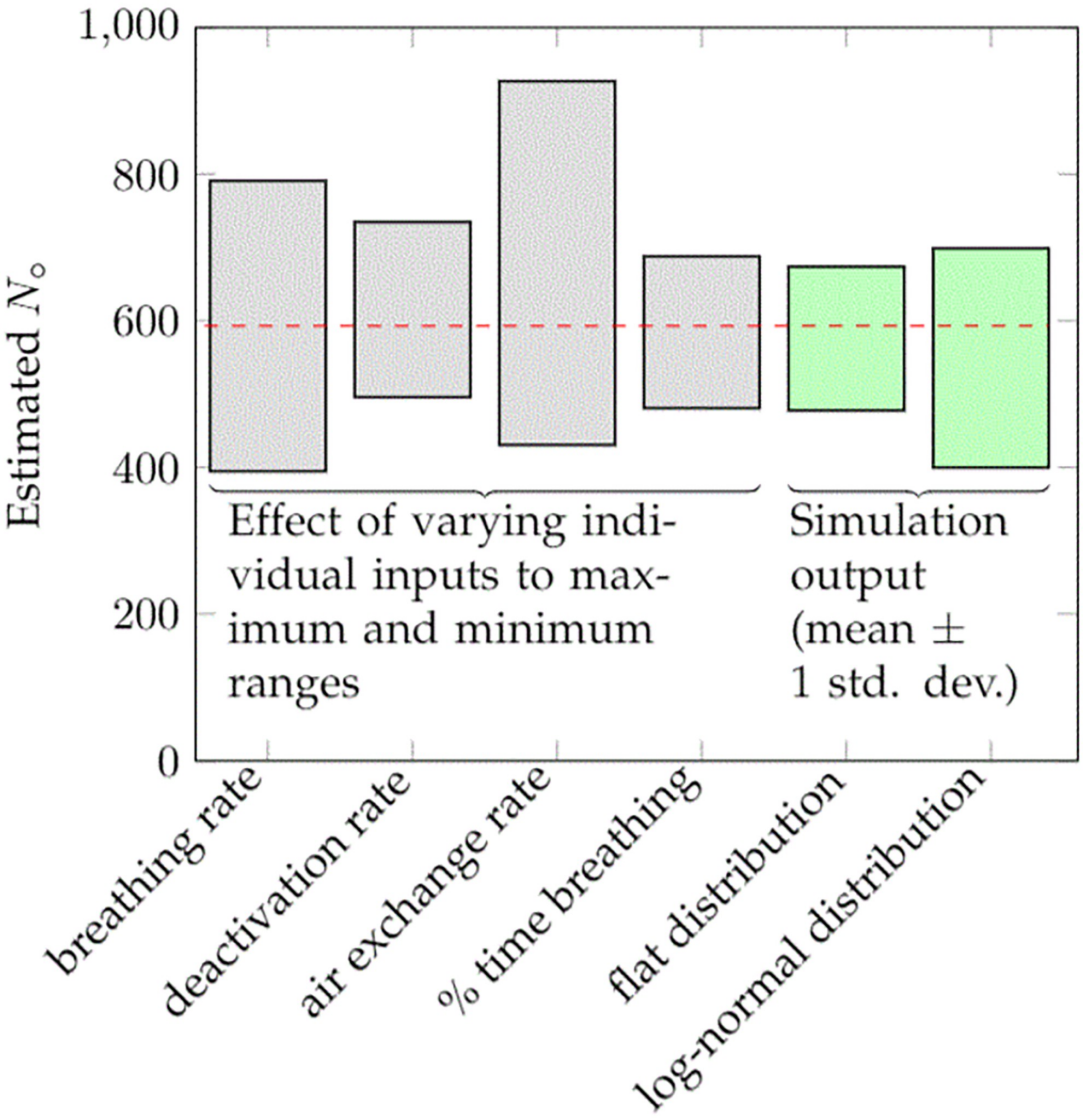
Result of varying individual parameters across entire range (keeping all other parameters constant, left side) and Monte Carlo simulations assuming uniform and lognormal distributions.

Fig 5 gives a graphical representation of the results of the parametric study. In the base case, we previously found ${\hat{S}}_{\text{q,breathing}}=461\;\text{quanta/hr}$, ${\hat{S}}_{\text{q,talking}}=10\;\text{quanta/hr}$, and ${\hat{N}}_{\text{0}}=593$ virions (range 322–2,012). To build intuition, we consider first some statistically unlikely cases in which a single variable is systematically over- or underestimated across all 5 cases, leaving all other inputs unchanged (left side of Fig 5). In the case when all air exchange rates are varied by ± 67%, for example, we find ${\hat{S}}_{\text{q,talking}}$ varies between 295–634 quanta/hr, ${\hat{S}}_{\text{q,breathing}}$ between 6–14 quanta/hr, and ${\hat{N}}_{0}$ between 431–927 virions. Varying the breathing rate by ± 33%, again across all cases identically, gives ${\hat{S}}_{\text{q,talking}}=346–692\;\text{quanta/hr}$, ${\hat{S}}_{\text{q,breathing}}=8–15\;\text{quanta/hr}$, and ${\hat{N}}_{0}=395–791$ virions. These results are all on the same scale as those in the base case, with the breathing rate and air exchange rates having a relatively larger effect on the estimates than the other variables. As an alternative measure the relative importance of the inputs, we also calculated partial rank correlation coefficients (PRCC) [58] for the breathing rate, viral deactivation rate, air exchange rate, and percentage of time breathing, under the lognormal simulation described below. We find, respectively, PRCC of -0.62, 0.25, 0.55, and 0.36, consistent with the relative contributions from the single-variable sensitivities.

We performed Monte Carlo simulations in two ways. In the first, for each case, each of the four variables was separately drawn from a uniform distribution with the above ranges. In the second, the variables were drawn from a lognormal distribution with a standard deviation equal to the above ranges. The uniform simulation, averaged across realizations and the 5 cases, finds mean (± standard deviation) values of *S*_talking_, *S*_breathing_, *N*_0_, of 489 ± 85 quanta/hr, 11 ± 2 quanta/hr, and 576 ± 101 virions, respectively; the comparable figures for the lognormal distribution are, respectively, *S*_talking_ = 540±162 quanta/hr, *S*_breathing_ = 12±4 quanta/hr, and *N*_0_ = 550±150 virions (right side of Fig 5).

In each case, the mean of the simulation does not precisely match the result from the mean of the inputs since the quanta emission rates are not linear functions of the inputs. Nonetheless, the uncertainty due to lack of precise knowledge of the above input variables is not so large as to create unreasonable ranges for the outputs.

In Spreadsheet S1 Table in the Supporting Information, we also present calculations for some other cases considered but not used for the main estimation, usually due to an issue with the well-mixed approximation. For example, we show the case in which the entire Korean call center is considered (greater area and lower attack rate), which results in a similar *N*_0_ = 240 virions. In the Supporting Information, we also discuss the case of a restaurant in Guangzhou, which is included in the spreadsheet but not in the case studies due to measurement ambiguities in both the attack rate and the relevant room volume. The values of *N*_0_ for the restaurant case are broadly consistent with the five cases here, with a range of *N*_0_ of 499 to 2,415; the range results from the range in the attack rate and different treatments of the relevant volume. We also consider the case of a German meat packing plant which is a likely case of aerosol transmission, but where the attack rate has a strong spatial dependence (indicating the well-mixed approximation is likely invalid [29].

## Discussion

To test for reasonableness, we compare the value of *N*_0_ found here to those of other, more well-studied airborne viruses [59]. In particular, the values of *N_0_* (∼300–2,000 virions) here are similar to that of influenza. Our search of the literature finds that most measurements would indicate an *N_0_* range of ∼300–9,000 virions for influenza (by way of comparison, Killingley and Bischoff quote a range influenza *N_0_* of ∼350–1,700 virions and ∼130–2,800 virions, respectively [17, 18]. In the case of influenza A aerosol infection, the human ID_50_ (dose at which 50% of subjects become infected) is widely cited as 0.6–3.0 TCID_50_ (the viral load at which 50% of cells in culture are infected) [60]. However, the range of viral copies per TCID_50_ varies significantly and has been measured by Fabian *et al*. [61], Ward *et al*. [62], and Yang [16] to be 300, 1000, and 452–2,100 viral copies, respectively. That is, in the case of influenza A, the human ID50 is in the range of ∼200–6,000 copies, which would imply a *N*_0_ (= ID50/ln(2)) for influenza of ∼300–9,000 copies [63]. The estimated value of ${\hat{N}}_{\text{0}}∼600$ viral copies also suggests that, like influenza, the infective dose of SARS-CoV-2 is of order 1 PFU. Fears *et al*. measured the change in infectivity of aerosolized SARS-Cov-2 with time. That work reports both genome copies per unit volume and PFU per unit volume [54]. Over 5 measurements spanning ∼ 8 hours, the ratio of PFU to genome copies ranges from ∼20 to ∼1,100, with an average of ∼500 copies/PFU, on the order of the *N*_0_ range found here [64, 65].

The consistency of the *N_0_* estimates from the 5 cases—each of which was derived assuming the same viral density—is surprising in light of the extremely broad range of viral loads discussed previously (spanning ∼8–9 orders of magnitude). If, in the alternative, we assume that *N*_0_ is in the range of 100–1,000 virions and calculate *N*, and thus the viral load, for each scenario, the range of viral loads that results is 5.7–7.5 Log_10_ copies/mL, again much narrower than the total range of viral loads seen in the population (∼2–10 Log_10_ copies/mL). This observation suggests that the index patients in the five case studies carried viral loads in the ∼ 50–80% percentiles—above average, but not exceptionally so. More importantly, this consistency suggests that for the types of cases studied here—where an asymptomatic or mildly symptomatic person infects numerous others through airborne transmission—the range of viral loads may be much narrower than the range implied by population-wide measurements.

**Fig 6:**
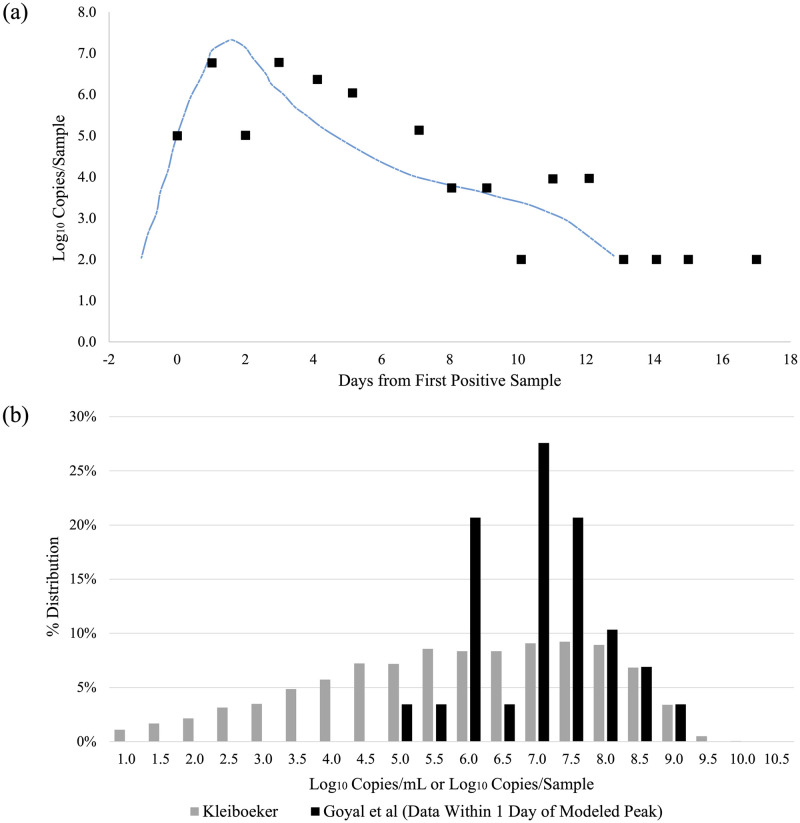
(a) Example Measured and Fit Viral Load Over Time (data and model shown from Patient S14 from Goyal *et al*.) [13]. The actual and modeled viral loads vary between ∼2 and 7 Log10 copies/sample across the entire period, but the modeled viral load for this patient lies in a narrower range of ∼6.3–7.3 Log10 copies/sample in the period which is ± 1 day around the time of peak viral load. (b) The grey bars are the distribution of viral loads (copies/mL) measured by Kleiboeker *et al*. The black bars are the distribution of viral loads (copies/sample) drawn from the 25 calibration datasets of Goyal *et al*., but only for the 29 data points taken within ± 1 day of the modeled peak viral load. The distribution of peak values, which may be more representative of viral load in index patients of our case studies, is significantly narrower than the Kleiboeker distribution.

Some recent studies have shown significant heterogeneity in viral loads of SARS-CoV-2 [66]. The relatively narrow range of relevant viral loads for superspreading events (whether through aerosols or other forms) found here, however, is consistent with longitudinal studies by Goyal *et al*. Specifically, it is well-established that patients with COVID-19 are most infectious within a window (∼ 1 day) of the onset of symptoms [8, 67]. Goyal *et al*. explored this phenomenon quantitatively in a set of two recent papers [13, 14]. In the first, the authors use longitudinal viral load data (i.e. repeated measurements on the same individual) from 25 patients to fit a compartmentalized viral load model. An example set of data and model fit (patient S14) from their work are shown in Fig 6(a) below: the model shows a very rapid increase of ∼5 orders of magnitude over a few days to a peak viral load of order 10^7^ copies/sample, a sharp drop of ∼2 orders of magnitude over the next two days, and a somewhat slower decline thereafter. However, in the window of ± 1 day around the peak viral load, the modeled load varies by only a factor of 10 (∼6.3–7.3 Log_10_ copies/sample) from patient to patient [13].

In the second analysis, Goyal *et al*. carry over the viral load model to analyze transmission dynamics in the population. This model finds that the time period in which infected patients are most likely to infect others (more precisely, to have a greater than 50% chance of infecting others), lies within a narrow 0.5–1.0 day period centered around the time of peak viral load [14].

Thus, there should be a significant difference between the viral loads that matter for transmission, which sample only a short time period around the peak viral load, and the viral loads measured by Kleiboeker and others, which sample all potential times after exposure. While the measured viral loads in the 25 longitudinal patient studies vary from ∼100 viral copies/sample (the approximate limit of detection [11, 12]) to ∼10^9^ copies/sample, the 25 models find only a very narrow range of fitted peak viral loads of ∼7.2–8.3 Log10 copies/sample. Goyal *et al*. note, however, that because there is more data during the period of viral decline, their fits are generally more reliable in this period [13, 14]. Alternatively, Fig 6(b) shows the distribution of the 29 instances of measured (not modeled) viral loads which fall within ± 1 day of Goyal *et al’*s modeled peak viral load (black bars), superimposed on the population-wide viral load measurement of Kleiboeker *et al*. (grey bars) [11, 13]. While this distribution of actual data is not as narrow as the range of modeled peak distributions, it is clearly far narrower than the population-wide distribution; ∼70% of the data lies in the range of 6.0–7.5 Log_10_ copies/sample. Interestingly, median viral loads for COVID-19 are consistent with those of a number of other respiratory viruses. Jacot *et al*., for example, report a median value of 6.77 Log_10_ copies/mL for SARS-CoV2 across 4,326 samples and compare SARS-CoV2 viral loads to those from 6,050 RT-PCR tests taken over 2015–2020 for 14 other respiratory viruses. All but two viruses show median viral loads between 5.76 and 6.83 Log_10_ copies/mL; for instance, influenza A and B have respective median loads of 6.01 and 6.83 Log_10_ copies/mL [10].

In sum, the period most relevant for infectivity samples only a narrow slice of the total range of viral loads observed across disease progression, helping to explain why selecting a single viral load value for the five cases results in a modest range for *N*_0_ values (∼300–2,000 virions).

### Risks of everyday activities

In a narrow sense, the value of *N*_0_ is not necessary for calculation of the risks of a given situation: one needs only ${\hat{S}}_{\text{q}}$, the quanta emitted per unit time. However, for assessing the risks associated with these quanta emission rates, averaged across the population, it matters greatly whether these quanta correspond to patients with typical peak viral loads, as we find in this work, or whether they correspond to patients with exceptionally high viral loads (i.e. “superspreaders”). Miller *et al*. calculate that, assuming that 1 quanta (*N*_0_ virions) is equal to 1000 viral RNA copies, the ∼1000 quanta/hour estimated in the Skagit choir case would correspond to a viral load of ∼10^11^ copies/mL [6]. Buonanno similarly calculates that a quanta emission rate of 142 quanta/hr during light exercise, assuming *N*_0_ = 50, corresponds to a viral load of 10^9^ copies/mL (so that if *N*_0_ = 1000, the viral load would be on the order of 10^10^ copies/mL [9]). Similarly, Evans assumes a viral load in saliva of 10^9^ copies/mL [48]. Bazant also finds *N*_0_ ∼ 10 virions under the assumption that the viral load is of order 10^9^ copies/mL [4]. However, in this work we associate similar quanta emission rates based on a viral load on the order of only 10^7^ copies/mL, which implies higher population-wide risks of aerosol transmission than these previous studies have suggested. We also emphasize that, consistent with Goyal’s work, the quanta emission rates here would apply only during a narrow window of ∼1 day around peak viral load; viral loads are at least an order of magnitude lower for times outside this range [14].

To illustrate the impact of the assumed viral load on the population-wide risks, consider a stylized case of a high-risk activity, where, based on the calibration quanta rates calculated from the case studies (e.g. 460 quanta/hour for talking, 10 quanta/hour for breathing), the risk of infection given the environmental conditions is 1/3 (i.e. exposure of *N* = 0.41*N*_0_). The details of the environment are not important for this purpose; rather, the environment and the quanta together are assumed to give rise to an infection probability of 1/3. Suppose that these calibration quanta emission rates correspond, as found here, to an index patient with a viral density of 10^7^ copies/mL. Across the population, the entire range of viral densities of ∼10^1^—10^10^ copies/mL will be represented, each with a different probability of causing infection. An individual with a viral density of 10^6^ copies/mL would, under the same environmental conditions, produce an exposure of 0.041 N_0_ and a risk of infection of 1-exp(-0.041) ∼ 4%; similarly, an index patient with a viral density of 10^8^ copies/mL would produce an exposure of 4.1 N_0_ and a risk of infection of ∼98%. The population-wide risk of this particular activity assuming a random index patient can be estimated by weighting these differing infection probabilities by the distribution of viral loads present in infected individuals. If the calibration quanta rate corresponds to a viral density of 10^7^ copies/mL, and the risk of infection for a person with this viral density is 33%, then the population-wide risk (averaging across the distribution of Kleiboeker *et al*.) is calculated to be 30.8%. That is, if the viral density for the calibration quanta is ∼10^7^ copies/mL, the quanta based calculation would be a good representation of the population-wide risk of this activity. On the other hand, if the calibration quanta rates were derived from an index patient with a viral density of 10^9^ copies/mL, the population wide risks (again averaging across the distribution of Kleiboeker *et al*.) would be only 2.9%—an order of magnitude lower—because relatively few individuals have viral densities as high as 10^9^ copies/mL.

To underscore this point, Table 3 shows the population-wide risks for the activity for different values of the viral density corresponding to the calibration quanta rate, assuming an infection risk of 1/3 for the calibration quanta rate. The table shows the percentile of the viral density distribution for each value of the viral density using the distribution of Kleiboeker *et al*. (e.g. 7.0 Log_10_ copies/mL = 71st percentile), the population-wide risk of infection (e.g. 30.8%), and the ratio of this population-based risk of infection to the risk calculated based on the quanta (e.g. 30.8%/33% ∼ 0.92). Previously, we showed that a range of *N*_0_ of 100–1,000 viral copies would, in the five case studies, imply a viral load of 5.7–7.5 Log_10_ copies/mL. Across this range, the order of magnitude of population wide risks (21.8%-∼50%) is similar to the quanta based risk of 33%. However, if the calibration quanta rates arise from high viral load individuals (10^9^-10^10^ copies/ mL), as has been suggested in earlier works, the population-wide average risk is only 0.4%-2.9%.

**Table 3:** Population wide-risks of infection assuming quanta-based risk of 1/3, for different values of the viral density corresponding to the calibration quanta rate. The risk of infection is assumed to be 1/3 at a given viral density (copies/mL) and then averaged across the distribution of viral densities measured by Kleiboeker *et al*. to derive the population-wide risk of infection.

	Log_10_ copies/mL @ Calibration Quanta Rate									
	5.5	6.0	6.5	7.0	7.5	8.0	8.5	9.0	9.5	10.0
Percentile of viral density distribution (%)	45.2	53.6	61.9	71.0	80.2	89.1	96.0	99.4	99.9	∼100
Population-wide risk of infection (%)	56.4	48.2	39.6	30.8	21.8	13.5	6.9	2.9	1.1	0.4
Ratio to quanta-based rate of infection	1.69	1.45	1.19	0.92	0.65	0.40	0.21	0.09	0.03	0.01

In the Supporting Information we detail the inputs and results of applying the quanta rates to various activities. The results would only be quantitatively applicable to the original SARS-CoV-2 strain, as the currently dominant variants have higher transmission rates (whether due to greater viral loads or smaller *N*_0_ is unknown). We emphasize, however, that it is easy to generate a factor of 10 or more difference in risks through the use of masks, filtration, or increased talking (which we estimate emits ∼50 times as many aerosols as breathing). For instance, in the good vs. bad classroom scenario, we assume the same volume, percentage of time talking, and total time in the classroom in both cases, but requiring masks and having strong air exchange/filtration changes the risk by a factor of 20, from 27% to 1%.

## Uncertainties and limitations

Our calculations are subject to numerous limitations and uncertainties. Our results for *S*_q_ depend only on parameters that are fairly consistent in the literature, and indeed our extracted values are consistent with previously published values for *S*_q_ [4, 7]; however, our calculated *N*_0_ values depend on parameters that still vary widely in the literature (e.g. the volume of emitted aerosols). As future research narrows that range of values, confidence in extracted *N*_0_ values will increase. Additional uncertainties include the following: (1) it is unknown to what degree close-range (droplet) transmission contributed to observed attack rates; if the attack rate due to aerosols is substantially lower, then the values of *N*_0_ would be higher than estimated here. (2) Though we have cross-checked our emitted volumes for talking and breathing across multiple measurements, the literature includes wide variations in measured values of $\bar{\phi }$. Reducing the uncertainty in $\bar{\phi }$ for various activities would inform the interpretation of the quanta calculated here: if $\bar{\phi }$ is much smaller than the values used here, the viral loads from the index patients studied would be at the high end of measured loads; if so, the average risks of aerosol transmission would be much lower than those calculated here, since very few patients would have these high viral loads. (3) Our calculations of viral loads are based on counts from RT-PCR. The particular specimen collected (sputum, NP swab, OP swab) may not directly probe the main source of aerosolized respiratory fluid, and more fundamentally, RT-PCR does not distinguish between infectious virus and fragments or non-viable virus particles. Measurements that probe the relationship between Ct values for RT-PCR and the amount of replication competent virus exhaled as a function of time during the course of COVID-19 infections for particular individual patients could enhance public health by honing quarantine rules, improving guidelines for risk reduction, and informing early treatment choices.

## Conclusions

A simple physical model can provide actionable information about viral transmission if, for a given activity, the model extracts consistent *N*_0_ values, or equivalently, consistent single-activity quanta emission rates, from different events with a range of durations and venue sizes. The validity of the information derived from the model increases with the number of events considered and the variation in the parameters for the different events.

If future studies find that the airborne model does not derive similar parameters from multiple events, that does not necessarily mean that transmission is not dominantly airborne. We suggest that researchers consider all of the following explanations for model failure: 1. The virus is not dominantly airborne; 2. The infectious viral loads of index patients are not similar; 3. Non-homogeneous distributions of immune responses could result in very different airborne transmission at different events even if all index patients had similar viral loads; 4. Transmission is so sensitive to a poorly measured values (e.g. air handling rate that the uncertainty in those measured values spreads the derived parameters so much that the values for different events do not seem similar. In contrast,if the airborne model does derive similar parameters from multiple events, then predictions from the airborne model can be used to calculate *N*_0_ and evaluate infection risks at future events.

For the five COVID-19 superspreading events considered in this study, the duration of the events and the volumes of the venues varied by more than an order of magnitude; however, quanta emission rates for talking or breathing extracted using the simple airborne model were similar across cases. This suggests that this simple model can be useful for informing decisions regarding risks of viral transmission, even for venues whose parameters are not precisely characterized. Of course, more precise predictions might be possible if the range of exhalation parameters extracted by the model was smaller and the parameters characterizing ventilation, the viral attenuation and settling rates were better known.

The consistency of the derived exhalation rates allowed us to calculate *N*_0_, which determines the probability of infection for a person exposed to *N* virions, assuming a Wells-Riley susceptibility. We also assumed that the rate at which the infected person is expelling virions is $\rho \bar{\phi }$, the product of concentration of replication competent virions in a volume of exhaled fluid *ρ* and the volume of fluid $\bar{\phi }$ exhaled per time. We used previously published results to calculate *ρ* and $\bar{\phi }$. Using those values in the airborne transmission model, we separately obtain *N*_0_ values for each of the five cases and find *N*_0_ values ranging from 300–2,000 virions, despite air volume variations of a factor of ∼30 and exposure time variations of a factor of approximately ∼10. If the range of $\bar{\phi }$ is narrow, a narrow range in *N*_0_ is a signature of relatively low dispersion in the viral loads of the index patients considered here. A low dispersion in the viral load of index patients is consistent with measurements of viral load as a function of time indicating that peak infectivity occurs during a very short time near the onset of symptoms when the viral load is a maximum that falls within a fairly narrow range [13, 14].

Interestingly, the *N*_0_ values for infection are similar for COVID-19 and influenza, which also has airborne transmission [10, 68]; however, that similarity does not imply that the transmission of the two viruses is similar. For instance, COVID-19 transmission is most probable during a very narrow time window (∼ 1 day) just before or at the onset of symptoms, whereas for many viruses, including influenza, transmission is dominated by symptomatic patients who may remain infectious for days [13, 14]. When comparing the spread of COVID-19 with the spread of other viral infections it is also important to note that transmission of viruses is greatly reduced if there is significant herd immunity. Thus, we propose that “enhanced transmission” due to an index patient with an average viral load who is just becoming symptomatic may result from a combination of the following: (1) a population with no previous exposure to the virus; and (2) confinement of the index patient with a group of people in a space that accumulates airborne particles (amplified by poor ventilation, vigorous activity, or lack of masks). Importantly, we find that the required confinement period may be as short as an hour and that enhanced transmission can occur at events attended by only a small number of people, such as family dinners. Furthermore, our conclusion that superspreading events can be adequately explained by index individuals with normal viral loads, while prior works analyzing some of the cases considered here have estimated the viral loads of index individuals to be much higher than typical [4, 6, 48].

In sum, our application of a simple aerosol transmission model to five well characterized transmission events suggests that transmission is airborne and that the index patients had viral loads typical of patients at the onset of symptoms; therefore, superspreading events do not require a superspreader. We have verified that the airborne model can be used to provide actionable information on risks of infectivity. Finally, we note that this work suggests that as new viruses or new variants of COVID emerge airborne transmission can be tested by applying a simple physical model if there is available information on infectivity rates, venue volumes, and exposure times.

## Supporting information

S1 Fig Viral load distribution and assumed viral density *ρ*. (TIF)

S1 Text Additional methodological detail. (PDF)

S2 Text Discussion of direct SARS-CoV-2 exhalation measurements. (PDF)

S3 Text Risks of everyday activities. (PDF)

S4 Text Guangzhou restaurant. (PDF)

S1 Table Supporting information calculations. (XLSX)
