# Supplementary figures and images for: Finding the infectious dose for COVID-19 by applying an airborne-transmission model to superspreader events
Source: PLoS One. 2022 Jun 9;17(6):e0265816. doi: 10.1371/journal.pone.0265816 (PMC9182663; doi:10.1371/journal.pone.0265816)

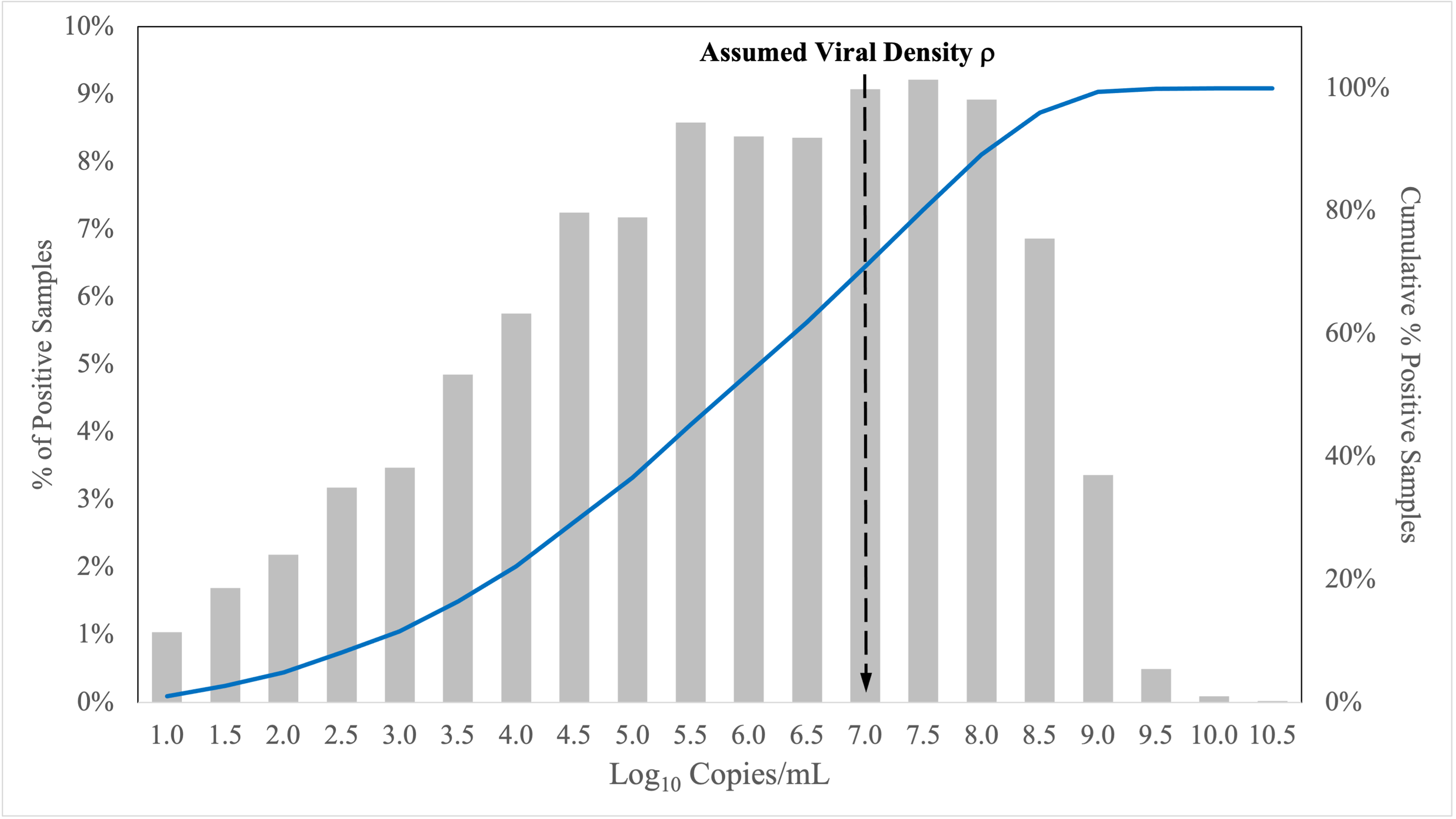

Supplement: S1 Fig — (TIF) [file pone.0265816.s001.tif]
