# Supplementary material for: Finding the infectious dose for COVID-19 by applying an airborne-transmission model to superspreader events
Source: PLoS One. 2022 Jun 9;17(6):e0265816. doi: 10.1371/journal.pone.0265816 (PMC9182663; doi:10.1371/journal.pone.0265816)
Supplement: S1 Text — (PDF) [file pone.0265816.s002.pdf]

S1: Additional Methodological Detail

Here, we give additional detail regarding the model and its inputs. We begin with a discussion of the airborne transmission model and derive the expressions shown in Fig 1 of the main text. We then review the literature relevant to our choices of input parameters, with a particular focus on the viral density ρ and the volume of aerosols emitted per unit time ϕ. We conclude by presenting a case in which the model closely reproduces observed airborne concentrations in a Singapore hospital.

Airborne Transmission Model

Fig 1 in the main text illustrates a standard "box" model of air flow that can be applied to aerosolized viral spread under appropriate assumptions, including SARS-Cov-2 by other authors [1–3]. Using this model, we derive q, the quanta emitted by the index patients, as they depend on breathing rate B, exposure time T, air or virion removal rate λ, and room volume V. By comparing the results of this model to estimates of q based on the Wells-Riley exposure model, we are able to derive an expression for $\bar{S}_{\mathrm{q}}$, the quanta emission rate of the index patient. (The bar notation $\bar{S}_q$ denotes a time-average: in the cases considered, the index patient is neither breathing nor talking/singing for the entire period, and $\bar{S}_{\mathrm{q}}$ averages across these activities during the event). This result permits estimation of the infection rate based solely on knowledge of the physical parameters of a hypothetical future scenario. However, it can also be combined with the virus-exhalation rate model presented below to reveal the viral dose threshold N_0.

Fig 1(a) illustrates the model principle and key parameters: an index patient releases a volume of aerosols per unit time at $\bar{\phi}$, and each aerosol contains ρ virions/unit volume. Equivalently, the index patient releases infectious quanta at a rate $\bar{S}_q$, where, by definition of a quanta, $\rho \cdot \bar{\phi} = \bar{S}_q N_0$. Air exchange and other factors cause infectious virus to decay at a rate λ. The overall concentration of virions is governed by a dimensionless dilution factor G, which is a function of the virion decay rate λ, the room volume V, and an interaction time T. Susceptible individuals breathe in virus-laden air at a rate B, resulting in a fraction r of them becoming infected. Fig 2 shows how each case's physical or epidemiological parameters (V, G, B, T, r) give a value of $\bar{S}_q$ for that case, which are averaged across cases to give an estimate of the emission rate $\hat{S}_q$ used to estimate the infection rate $\hat{r}$ in a hypothetical scenario.

Given the concentration (virions/unit volume of air) of virions $C(t)$ and inhalation at a constant breathing rate B (volume/unit time breathed in/out) for a total time T, the total number N of virions breathed in is $N = B \int_0^T dt\, C(t)$. The concentration C depends on both spatial coordinates and time. It is common

in indoor air quality calculations to make the simplifying assumption that the air is "well-mixed" – i.e. that the virions are spread evenly across the volume in the room so that one can neglect the spatial dependence. This assumption is justified if the time for virions to spread across the room is small compared to the timescale of the decay processes above due to air exchange and other virion losses. The mixing time for cough-generated aerosols has been characterized, where a coughing simulator was placed on one end of a 2.7 m × 2.7 m × 2.4 m environmental chamber, and the concentration of small aerosols (0.3-4.0 µm) was measured as a function of time at various locations [4]. The measurement found that after about 5 minutes, the concentration reached a steady state which was uniform across the room. Note also that in the experiment, the chamber was sealed during the coughing simulation (zero air exchanges per hour) so that there was no airflow that would cause additional mixing.

Within the well-mixed approximation, we can calculate the number of virions $n(t)$ over time. We assume that the person emits $\bar{S}$ new virions per unit time, and that since the air is well-mixed, the number of virions lost to decay per unit time is $\lambda \cdot n(t)$, where λ is a constant which can be the sum of multiple distinct contributions e.g. fresh air exchange, viral inactivation, as discussed in more detail below. We have that $n(t)$ obeys

$$\frac{dn}{dt} = \bar{S} - \lambda n(t)$$

which has the usual solution of a transient term decaying at a rate λ toward a steady state $n_{eq} = \bar{S}/\lambda$:

$$n(t) = n_{eq} + (n(0) - n_{eq})\, e^{-\lambda t}$$

In the cases we consider, we assume that the environment starts off "clean" – i.e. that $n(0) = 0$. That is, we assume there are no viral particles in the air before the choir assembles, or before the riders enter the bus, etc. In this case, we have

$$n(t) = n_{eq}\left(1 - e^{-\lambda t}\right)$$

Since the concentration $C = n\ /\ V$ where V is the volume of the room, we can solve for the total inhaled particles N over a time T (where B is the breathing rate i.e. the volume inhaled per unit time).

$$N = B \int_0^T dt C(t) = BTC_{\text{eq}} \left(1 - \frac{1}{\lambda T}\left(1 - e^{-\lambda T}\right)\right) \tag{1}$$

Here C_{eq} is the steady-state (equilibrium) concentration of virions in the space.

$$C_{eq} = \frac{n_{eq}}{V} = \frac{\bar{S}}{\lambda V}$$

It is convenient to rewrite the expression for N in terms of a dimensionless attenuation factor G which expresses the net effect of viral removal, where

$$G = \lambda T (1 - \frac{1}{\lambda T}(1 - e^{-\lambda T}))^{-1} \tag{2}$$

We can then write N more compactly as the product of the total virions exhaled ($\bar{S}T$) and the fraction of the total room volume (adjusted for G) that the susceptible patients inhale (BT/GV),

$$N = \bar{S}T \cdot \frac{BT}{GV} \tag{3}$$

From (3), we can derive expressions for either $\bar{S}_{\mathrm{q}}$ or N_0. For $\bar{S}_q$, recall that the inhaled quanta $q = N/N_0$ and that $\bar{S}_q = \bar{S}/N_0$. Further, per the Wells-Riley model, $q = \ln(1-r)$, so that dividing both sides of (3) by N_0, we have

$$q = -\ln(1-r) = \bar{S}_{\mathrm{q}} T \cdot \frac{BT}{GV} \tag{4}$$

This equation links the infection rate (the first equality) to the properties of the spaces/rooms (the second equality). This equality then permits us to solve for the source strength q: (1) via the infection rate (the first equality); and (2) via detailed analysis of the spaces/rooms in each case. The second equality permits us to solve for the source strength $\bar{S}_{\mathrm{q}}$, yielding,

$$\bar{S}_{\mathrm{q}} = -\frac{\ln(1-r)}{T} \cdot \frac{GV}{BT} \tag{5}$$

which tells us that the source strength is equal to the quanta inhaled per unit time multiplied by the ratio of the room volume (adjusted by a factor G), to the volume of air inhaled. Equivalently, in (3), writing $N = -N_0 \ln(1-r)$, we have

$$N_0 = -\frac{1}{\ln(1-r)} \cdot \bar{S}T \cdot \frac{BT}{GV} \tag{6}$$

which is the second key equation in this work.

Discussion of Model Inputs

Breathing rate B: The value of the breathing rate B has been well-characterized in a number of studies and is typically assumed to be in the range of 0.45 m^3/hr - 0.60 m^3/hr for sedentary activities [5,6]. Relevant for this analysis is the measurement of Binazzi *et al.*, which found that quiet breathing, reading with a normal voice, and singing all had similar breathing rates (0.54 ± 0.21 m^3/hr, 0.54 ± 0.21 m^3/hr, and 0.61 ± 0.40 m^3/hr, respectively) [7]. The breathing rate rises to $\sim$1.3-1.5 m^3/hr during "moderate" activities (easy cycling, climbing stairs) and $\sim$2.5-3.3 m^3/hr during "heavy" activities (cross country skiing, climbing with load) [8]. For purposes of this analysis, we use B = 0.5 m^3/hr for the choir, call center, and bus cases, and B = 2.0 m^3/hr for the fitness center case.

Decay rate λ: As noted above, λ is the result of several contributions that sum to give the total rate, namely:

- λ_{air}= air exchange rate = rate at which fresh air replaces stale air in the room. We use the EPA recommended λ_{air}= 1.5 air changes per hour for nonresidential structures for the choir, office, and fitness center cases [9], and 3.0 air changes/hour for the bus cases based on the measurements of Tong *et al.* [10] [11]. We discuss in the main text the effect of varying these values to take into account uncertainties such as the difference between structures in Asia and those in the U.S.

- $\lambda_{\text{deactivation}}$ = decay rate of SARS-CoV-2 in air, due to both settling and inactivation in air. For settling, Diapouli *et al.* report aerosol settling rates in indoor settings which average approximately 0.3 hr^{-1}: PM 2.5 decay rates ranged from $\sim$0.1-0.4 hr^{-1}, while measured PM 10 decay rates range from $\sim$0.05-0.65 hr^{-1} [12]. For SARS-CoV-2 viral deactivation, both van Doremalen *et al.* and Fears *et al.* measured the decay in infective virus in a Goldberg drum, which in principle eliminates the effects of settling. However, van Doremalen measured a half-life of SARS-Cov-2 1.09 hours in air (λ_2= 0.64 hr^{-1}) [13], while Fears *et al.* were not able to detect meaningful viral inactivation of 2-3 μm SARS-CoV-2 aerosols over a 16 hour period (i.e. measured $\lambda_{\text{deactivation}}$= 0) [14] [15]. For purposes of this calculation, we include a contribution of 0.3 hr^{-1} due to settling and a contribution of 0.32 hr^{-1} due to inactivation (averaging the van Doremalen and Fears measurements) for a total value of $\lambda_{\text{deactivation}}$ = 0.62 hr^{-1}.

- λ_{filter} = effect of filtration. In the case studies considered, there is no filtration; this parameter is only used in the scenario analysis.

Viral density ρ**:** Across a sample of $\sim$ 3,300 patients testing positive in Germany, Jones *et al.* measured a mean viral load of 5.7 Log_{10} copies/mL with an estimated standard deviation of $\sim$1.8 Log_{10} copies/mL; nearly all of these tests relied on nasopharyngeal and oropharnygeal swabs [16]. Similarly, in a sample of all 4,428 positive RT-PCR tests (defined as a cycle threshold value for RT-PCR $\leq$ 38.0), Kleiboeker *et al.* measured a mean and median, respectively, of 5.85 and 6.05 log_{10} copies/mL, with a range of 0.91-10.42 Log_{10} copies/mL, 15.3% results greater than 8 log_{10} copies/mL, and an estimated standard deviation of $\sim$2.0 log_{10} copies/mL (Fig S1) [17] [18]. Other measurements include Arnaout *et al.* (initial positive results from 4,774 patients, mean of $\sim$5.2 log_{10} copies/mL) and Jacot *et al.* (initial positive results from 4,172 patients taken >99.5 % by NP swab, mean of $\sim$6.5 log_{10} copies/mL, and median of 6.77 log_{10} copies/mL) [19,20]. Arnaout finds a maximum viral load of 2.5$\times$ 10^9 copies/mL (9.4 log_{10} copies/mL) while Jacot finds a maximum viral load of $\sim$2 $\times$ 10^{10} copies/mL (10.3 log_{10} copies/mL), broadly consistent with Kleibocker and Jones. In all of these measurements, the probability of a given viral density drops sharply beginning at $\sim 10^8$ copies/mL.

Figure S1: **Viral Load Distribution (Kleibocker *et al.*) and Assumed Viral Density** ρ**. Viral load distribution (copies/mL) as measured by Kleibocker *et al.*. Viral loads were derived from all 4,428 positive test results (cycle threshold value for RT-PCR $\leq$ 38.0) in a U.S. testing laboratory; values include all viral copies, some of which may not be infective. Viral load is referenced to the left hand axis and shows a peak near 7.5 log_{10} copies/mL, near the assumed value of ρ. The cumulative sum of the Kleibocker et al. distribution is also shown, in reference to the right hand axis.**

There are a few subtleties in interpreting this data. First, the remarkable breadth of the distribution -- spanning 9 orders of magnitude in viral titre -- needs to be interpreted carefully. The PCR tests underlying the distribution were taken at different points after infection, so the wide range of viral loads arises from not only inhomogeneity across patients, but also, changes during disease progression [21]. Said differently, a single patient tested continuously from exposure to recovery would show a range of viral loads from the limit of detection ($\sim$100 copies/mL) to the peak viral load (conceivably 10^7-10^9 copies/mL). However, as discussed in the main text, Goyal *et al.* find that at peak infectiousness (the relevant range for this work), the distribution of viral loads is much narrower.

Second, although it has been shown that infectiousness strongly correlates to viral density at a fixed point in disease progression [22], not all virions detected by RT-PCR are infectious, and the fraction of infectious virions may change over time or depend in a nonlinear way on the viral density [23]. For the superspreading events we considered, the index patients were either pre-symptomatic or just beginning

to experience symptoms. Modeling work by Ke *et al.* suggests that at representative peak viral loads of 10^7 copies/mL, 20-90% (i.e. order 1) of virions are infective , but a much smaller fraction (10% or less) is infective at lower viral loads of e.g. 10^4 copies/mL [23]; these lower viral loads would present themselves after peak infectivity. These results are consistent with laboratory results, where infectious virus can be cultured only shortly after symptom onset [24–29]. Bullard *et al.* found that $\sim$ 30-80 % of of samples in days 1-5 after developing symptoms (mean Ct values $\sim$20-30) yielded positive cell cultures, but that no samples after day 8 yielded positive cell cultures [26]. Similarly, Wölfel *et al.* were able to recover live virus from 17% of swab samples and 83% of sputum samples in the first week of symptoms but not thereafter [25]. Similar results are observed in hamsters and ferrets [30,31]. To the extent that we considered index patients at the onset of symptoms when the viral loads detected by PCR are a maximum [32, 33], infectious virions will be on the order of total virions. We therefore assume that viral loads for the index patients are characterized by the narrow range of viral load peaks found using PCR, and that those loads represent competent virus; however, we note that later in the course of the illness this approximation would be enormously inaccurate [34]. Finally, we emphasize that the narrow range of $S_{\mathrm{q\ (breathing,\ talking)}}$ values that we found supports the suggestion that the viral load of replication competent virus was similar for all index patients.

Finally, some care must be taken in translating viral loads measured by NP swab to viral loads in aerosols. The NP swab sample is placed into viral transport medium, and the number of copies/mL reported by RT-PCR includes the effect of this dilution: that is, the viral density in the fluid sampled from the nasopharynx is greater than the measured viral density from the viral transport medium. However, in the cases studied here, viral emission is dominated by talking (or singing), for which aerosolized particles originate from the laryngeal region, which has a different and lower viral density than the nasopharynx [35–39]. As discussed in detail below these two effects are approximately offsetting.

Discussion of Viral Loads in Swabs

The value of the viral density ρ represents the number of viral copies/mL in emitted aerosols. Reported viral copies/mL – from nasopharyngeal (NP) or oropharyngeal (OP) swabs, saliva, or sputum – can differ from ρ for at least two reasons: first, the sample itself may or may not be diluted prior to a copies/mL measurement; and second, the sample may or may not be taken from a part of the respiratory system that is relevant to ρ.

On the issue of dilution, sputum and saliva samples are frequently measured in native conditions [25,

40], while NP and OP swabs are typically placed into 1-3mL of viral transport medium [19,25,38]. Swabs are usually left in place for several seconds to absorb secretions [41,42], and the absorption capacity is typically of order 100-150 μL [43]. This suggests a dilution of order 10-30x of the nasopharyngeal/oropharyngeal fluid by the viral transport medium.

On the issue of the relevant part of the respiratory system, aerosols generated by different activities originate from different parts of the respiratory system, which may in turn have different viral loads [35–37]. Johnson *et al.* measured the particle size distributions during breathing, speaking, and coughing, and found three distinct modes with different origins [35–37]: (1) breathing produced aerosols with a median diameter of 0.8 μm. These particles are believed to be generated in the bronchioles during closing and reopening of small airways. (2) Speaking and coughing created similar sized aerosols (median diameter of 1.0 μm), but these aerosols originate in the laryngeal region; Johnson *et al.* specifically tested for this effect using unmodulated vocalization in order to avoid the effect of large oral particles. (3) Speaking and coughing also created large particles (median diameter 200 μm) which were confirmed via dye to originate from saliva.

In the situations analyzed here, because ϕ_{talking} is approximately 50 times larger than $\phi_{\text{breathing}}$, the second mode (small aerosols due to speaking) will dominate airborne viral transport. To the extent that these aerosols arise from the laryngeal region, oropharyngeal swabs are likely to be more representative of viral densities than nasopharyngeal swabs. In turn, various measurements have shown that oropharyngeal swabs have significantly lower viral loads than nasopharyngeal swabs:

- Hernes *et al.* performed PCR measurements of contemporaneous NP and OP swabs from 32 patients with influenza. Both swabs were diluted in the same volume of viral transport medium, so a comparison of cycle threshold values gives a direct measurement of the relative viral loads in the nasopharynx and oropharynx. On average, NP swabs had 54 times higher viral loads than OP swabs, with a ratio of 23 times for Influenza A and 80 times for influenza B [38].

- Hernes *et al.* performed a similar set of measurements on 19 elderly patients, testing for a range of respiratory viruses through PCR. On average, across patients and viruses, the viral load was 19 times higher in NP swabs than in OP swabs [39].

- Wang *et al.* compared 120 matched NP/OP swab pairs for SARS-CoV-2 patients, of which 57 were positive by either NP or OP swabs ("true positives") [42]. Of these 57 patients, 52 had a lower Ct value (higher viral load) for NP swabs versus OP swabs. The mean Ct value of NP swabs was 35.3

(95% CI 33.9-36.8), while that of OP swabs was 38.7 (95% CI 37.7-39.6). The difference in mean Ct values of 3.4 corresponds to a factor of $\sim 2^{3.4} \sim 10$ greater viral load in NP swabs than OP swabs, not dissimilar to the Hernes measurements.

The greater viral load in the nasopharynx versus the oropharynx ($\sim$ 10-50x) is therefore (for unrelated reasons) approximately offsetting to the dilution of NP swabs by viral transport medium ($\sim$ 10-30x). As a side note, we have assumed in these calculations that the difference in quanta emission rates in speaking and breathing are due only to differences in emitted aerosol volumes. However, viral densities in sputum/the lower respiratory system (breathing) are generally thought to be higher than in the oropharynx (speaking) [25, 44], so the difference between breathing and speaking may be narrower than the factor of $\sim$46 we find here.

Volume expelled per unit time ϕ: We first discuss the relative contributions of talking, breathing, and singing, and then the differences in measurements of ϕ in the literature.

- *Talking*: Stadnytskyi *et al.* recently measured aerosol volumes by scattering of laser light off droplets generated during repetition of the phrase "stay healthy" for 25 seconds [45]. The study infers a dehydrated droplet diameter of $\sim$4 μm and a hydrated droplet diameter $\sim$12-21 μm, corresponding to hydrated volumes of 60 to 320 nL. We use the average of these two values – i.e. when talking, ϕ_{talking} = 190 nL/25s = 7.6 nL/s = 2.7 $\times 10^{-2}$ mL/hr.
- *Breathing only*: Morawska *et al.* measured differences in expelled particle density when only breathing and when talking (e.g. saying "aah" and counting) [46]. Averaging across the talking and quiet breathing scenarios, these measurements show that talking releases about an order of magnitude more particles by number than does breathing while quiet, but because vocalization releases larger particles, the volume ratio of talking to only breathing is higher, at $\sim$45.7× [47]. Thus, for breathing we use $\phi_{\text{breathing}}$ = 1/45.7x 7.6 nL/s $\sim$ 0.2 nL/s = 6.0 $\times 10^{-4}$ mL/hr.
- *Singing*: Singing expels about 6 times as many particles as talking [48, 49], so for this case we use ϕ_{singing} = 6 x 7.6 nL/s = 45.6 nL/s. = = 1.6 $\times 10^{-1}$ mL/hr.

The order of magnitude of the volume of aerosols emitted during talking varies significantly in the literature depending on the measurement method used. For instance, Miller *et al.* estimate, based on the work of Morawska, that speaking results in emission of $\sim$1-10 nL of aerosols per hour [3, 46], while Evans

estimates that breathing and speaking emit 60 and 600 nL/hour, respectively [50]. In contrast, Stadnytskyi *et al.* find volumes of 60-320 nL over just 25 seconds of speaking -- some 3-4 orders of magnitude higher. This large difference may be due partially to the differences in particle sizes measured. Stadnytskyi *et al.* estimate that the aerodynamic particle sizer (APS) method used by Morawska *et al.* may measure particles of hydrated diameter ∼8.7 µm and less, which are outside of the 12-21 µm range in the scattering experiment [45]. Nonetheless, a sharp drop of 3-4 orders of magnitude for a halving of the particle diameter is surprising. A second factor that may account for the difference is that the measurement of Morawska *et al.* uses an APS, which may not count all particles emitted. Asadi *et al.*, who perform similar measurements to Morawska using an APS, note that their reported particle emission rates are to be viewed in relative, not absolute terms [48].

We believe that Stadnytskyi's estimate is the more appropriate one to use here as it is corroborated by similar measurements as well as by exhaled breath condensate measurements. In the case of talking, Smith *et al.* recently performed a similar measurement to Stadnytskyi on 7 volunteers, measuring particle sizes for talking and coughing [51]. They found that talking emitted particles with a 1-10 µm range (average diameter of 4 µm), and that a "superemitter" with ∼ 17 times the volumes of those of other volunteers emitted 0.003 ± 0.001 g when saying "stay healthy" 10 times. Assuming a 15 second speaking time and an aerosol density equal to that of water, this mass measurement corresponds to a typical (non-super-emitting) individual emitting 4.2×10^{-2} mL/hour while talking, similar to our estimate (based on Stadnytskyi's measurements) of $\phi_{\text{talking}} = 2.7 \times 10^{-2}$ mL/hr. A recent study of quanta emission rates across various airborne viruses has also adopted the volumes measured by Stadnytskyi *et al.* [52].

As a further cross-check, we can compare our computed value of $\phi_{\text{breathing}}$ to experiments that measured respiratory fluids using exhaled breath condensates (EBCs). In an EBC measurement, a patient breathes into a chilled tube which collects the condensed breath. That condensed breath can then be tested for volatile and non-volatile constituents, including pathogens [53–55]. Breath samples consist almost exclusively of water vapor from the lungs. The water vapor cannot carry the virus, but the breath samples also contain a small contribution of aerosols from respiratory fluids. Those aerosolized fluids can carry viruses, as well as other non-volatile condensates that are present in the respiratory system. Thus, EBC's include both condensed water vapor and condensed aerosols. Effros *et al.* compared the concentration of non-volatile solutes in condensed respiratory fluids to the concentration of the same solutes in airways. They found that the concentration of the non-volatile solutes in EBC's was reduced by an average factor of ∼20,000:1 (± ∼2,500:1) over the concentrations in lungs, with the reduction factor varying between ∼1000:1

to ~50,000:1 [53] [56]. If the non-volatile solute concentration in exhaled droplets is similar to concentrations in lungs, then respiratory aerosols account for ~1/20,000 of the total water loss through breathing. Water loss through breathing is well-characterized at ~350-400 mL/day (~16 mL/hr) [54, 57]. The dilution factor of 20,000 from EBC measurements would therefore imply aerosolized breath volumes of 16/20,000 = 8×10^{-4} mL/hr, consistent with our value of $\phi_{\text{breathing}}$ of $\sim 6 \times 10^{-4}$ mL/hr [58]. In a separate publication, Effros *et al.* estimate that ~4.5 nL of airway lining fluid is exhaled per liter of breath [55], which, assuming a breathing rate of 500 L/hr (0.5 m^3/hr), translates into $\sim 2.3 \times 10^{-3}$ mL/hr of emitted volume, within a factor of ~4 of our figure for $\phi_{\text{breathing}}$ of $\sim 6.0 \times 10^{-4}$ mL/hr. That is, the volume of breathing aerosols implied by Stadnytskyi's (and Smith's) speaking measurements and our assumed ratio of breathing/speaking volumes are consistent with the volume of breathing aerosols from EBC measurements.)

Check: Singapore Isolation Wards Calculation

As discussed at some length above, the model used here contains several assumptions – that the well-mixed model applies, that Ct values from NP swab can be used to infer viral densities for emission without further adjustment for dilution, and that the volumes emitted are in line with those measured by Smith and Stadnytski [45, 51]. To further check the reasonableness of these assumptions, we consider an interesting case study from Chia *et al.*, who measured SARS-CoV2 viral copies in the air of three airborne infection isolation rooms (AIIRs) in a Singapore hospital [59]. Compared to other measurements of SARS-CoV-2 in the air, the Chia study is notable because the patients are isolated and the study reports patient Ct levels measured by nasopharyngeal swab. The AIIR are estimated to have a volume of 50 m^3 and the air change rate is reported as 12/h; all three patients were reported to be coughing [59]. We can estimate the patient viral load from the Ct value, as well as the virion emission rate based on coughing, and compare calculated and measured virion concentrations in the room (virus copies/m^3 of air). This comparison provides a reasonableness check for using unadjusted NP-swab copies/mL values for the aerosol viral density ρ. The main steps of the calculation are as follows:

- Conversion of Ct value to copies/mL: Although the precise conversion of Ct to copies/mL will depend on the efficiency and limit of detection of each assay, in practice these values are often similar, such that conversions from different studies will give similar copies/mL values. For this calculation, we average the results of converting Ct to copies/mL using the Zou, Jones, and Jacot formulae (see the section "Discussion of Ma *et al.* Exhaled Breath Measurement" in the Supporting Information for

details); Goyal *et al.* follows a similar procedure and uses the Zou conversion in his analysis in cases when only the Ct value is reported [32]. The result is not highly sensitive to which of the three conversions is chosen: for instance, for patient 2, the range of viral loads is 1.1 $\times 10^8$ - 4.7 $\times 10^8$ copies/mL.

- Aerosolized volume emitted per hour: This is the product of coughs per hour and the volume of aerosols per cough.

  – We are not aware of any direct measurement (or reporting by Chia) of the cough frequency for SARS-Cov-2 patients, but in a sample of 142 influenza patients, Yan *et al.* measured a cough frequency of 33 coughs/30 minutes for male volunteers and 21 coughs/30 minutes for female volunteers – i.e. approximately 1 cough per minute [60], which is the value we assume here. As a reasonableness check, Zhang *et al.* assume 22 coughs/hour for their simulation of influenza transmission [61].

  – For the volume per cough, we can calculate from Morawska's measurements (which only consider aerosol particles) that a single cough releases $\sim$11 $\times$the volume of breathing for 1 minute; using our previously derived value of $\phi_{\text{breathing}}$= 6.0 $\times 10^{-4}$ mL/hr, we calculate that a single cough releases 1.1 $\times 10^{-4}$ mL of aerosolized particles. We emphasize that the total fluid volume due to a cough is much larger than this due to droplets, but we are only interested in aerosols. As a check, Smith *et al.* recently measured that a super-emitter, who emitted 17 times as much as other volunteers, released a mass of 0.07 $\pm$ 0.05 g of fluid per cough, of which 2 $\pm$1 percent was of aerosol size, and the remainder were large droplets (100-1000 μm). This implies an aerosolized volume of 8.2 $\times 10^{-5}$ mL per cough, very close to the 1.1 $\times 10^{-4}$ mL above.

  – Combining these values of 1.1 $\times 10^{-4}$ mL of aerosols/cough and a coughing rate of 1 per minute, we get a volume ϕ_{coughing} = 6.7 $\times 10^{-3}$ mL/hour.

- Concentration in room: We then can calculate the steady state concentration of viral copies in the room as $C = \rho\phi_{\text{coughing}} \ / \ \lambda V$, where λ = 12/hr and V = 50 m^3; the volume is derived from the stated exhaust flow rate of 580 m ^{3}/hr and the air changes of 12/hr. We compare this to the viral copies/m^3 found for the particle size range 1-4 μm reported by Chia *et al.* in the right two columns of Table S1. We emphasize that there are no adjustments for the NP swab number of viral copies/mL in the calculation; nonetheless, the calculated concentrations are quite close to the reported concentrations. That is, if we use the unadjusted copies/mL derived from nasopharyngeal swab, we are able to closely

reproduce, at least in this case, the observed airborne viral concentration. This calculation provides some support for the idea that the dilution of the NP swab due to the viral transport medium is approximately offset by the lower viral load of the oropharyngeal region versus the nasopharyngeal region.

Table S1: Estimated and Reported Airborne Concentrations in Singapore Isolation Rooms measured by Chia *et al.* [59]

Patient	Ct Value	ρ [copies/mL]	Calculated C [copies/m^3]	Measured C [copies/m^3]
1	33.22	8.1×10^3	<1	ND
2	18.45	2.0×10^8	2199	1384
3	20.11	6.3×10^7	707	927

References

[1] Buonanno G, Stabile L, Morawska L. Estimation of airborne viral emission: Quanta emission rate of SARS-CoV-2 for infection risk assessment. Environ Int. 2020;141:105794.

[2] Buonanno G, Morawska L, Stabile L. Quantitative assessment of the risk of airborne transmission of SARS-CoV-2 infection: prospective and retrospective applications. Environ Int. 2020;145:106112.

[3] Miller SL, Nazaroff WW, Jimenez JL, Boerstra A, Buonanno G, Dancer SJ, et al. Transmission of SARS-CoV-2 by inhalation of respiratory aerosol in the Skagit Valley Chorale superspreading event. Indoor Air. 2020;doi:10.1111/ina.12751.

[4] Lindsley WG, King WP, Thewlis RE, Reynolds JS, Panday K, Cao G, et al. Dispersion and exposure to a cough-generated aerosol in a simulated medical examination room. J Occup Environ Hyg. 2012;9(12):681–690.

[5] Augenbraun BL, Lasner ZD, Mitra D, Prabhu S, Raval S, Sawaoka H, et al. Assessment and mitigation of aerosol airborne SARS-CoV-2 transmission in laboratory and office environments. J Occup Environ Hyg. 2020;17(10):447–456.

[6] Adams WC. Measurement of breathing rate and volume in routinely performed daily activities. California Air Resources Board (Contract a033-205); 1993. Available from: https://ww3.arb.ca.gov/research/single-project.php?row_id=64931.

[7] Binazzi B, Lanini B, Bianchi R, Romagnoli I, Nerini M, Gigliotti F, et al. Breathing pattern and kinematics in normal subjects during speech, singing and loud whispering. Acta Physiol. 2006;186(3):233–246.

[8] U S EPA Office of Research and Development. Chapter 6 (Inhalation Rates). In: Exposure Factors Handbook. Washington DC 20460: U.S. Environmental Protection Agency; 2011.

[9] U S EPA Office of Research and Development. Chapter 19 (Building Characteristics). In: Exposure Factors Handbook. Washington DC 20460: U.S. Environmental Protection Agency; 2018.

[10] Tong Z, Li Y, Westerdahl D, Adamkiewicz G, Spengler JD. Exploring the effects of ventilation practices in mitigating in-vehicle exposure to traffic-related air pollutants in China. Environ Int. 2019;127:773–784.

[11] The event in the bus occurred during winter, so the bus windows were believed to be closed (Y. Shen, personal communication).

[12] Diapouli E, Chaloulakou A, Koutrakis P. Estimating the concentration of indoor particles of outdoor origin: a review. J Air Waste Manag Assoc. 2013;63(10):1113–1129.

[13] van Doremalen N, Bushmaker T, Morris DH, Holbrook MG, Gamble A, Williamson BN, et al. Aerosol and Surface Stability of SARS-CoV-2 as Compared with SARS-CoV-1. N Engl J Med. 2020;382(16):1564–1567.

[14] Fears AC, Klimstra WB, Duprex P, Hartman A, Weaver SC, Plante KS, et al. Persistence of severe acute respiratory syndrome coronavirus 2 in aerosol suspensions. Emerg Infect Dis. 2020;26(9):2168–2171.

[15] The source of this discrepancy is unclear, though it is possible that the measurement of van Doremalen includes some effect of settling. Fears *et al.* explicitly note that the rotation rate of the Goldberg drum used was sufficiently high to create a suspended aerosol [14]. Measurements by Huang *et al.* found a half-life of 1.38 ± 0.05 hours for 3 µm particles rotating at 5 rpm in a Goldberg drum, not dissimilar from the 1.09 hour half-life found by van Doremalen [62].

[16] Jones TC, Mühlemann B, Veith T, Biele G, Zuchowski M, Hoffmann J, et al. An analysis of SARS-CoV-2 viral load by patient age. medRxiv. 2020;(medrxiv;2020.06.08.20125484v1).

[17] Kleiboeker S, Cowden S, Grantham J, Nutt J, Tyler A, Berg A, et al. SARS-CoV-2 viral load assessment in respiratory samples. J Clin Virol. 2020;129:104439.

[18] The standard deviation for Jones *et al* is estimated from Table 2 in Jones [16]. Kleiboeker *et al.* do not report a standard deviation, so the figure of $\sim$2.0 $\log_{10}$ copies/mL was calculated based on the underlying data provided to us by Kleiboeker (private communication). The mean $\log_{10}$ copies/mL values for Arnaout and Jacot were calculated based on digitizing the data in their figures 2(a) and 1(a), respectively [19, 20].

[19] Arnaout R, Lee RA, Lee GR, Callahan C, Cheng A, Yen CF, et al. The Limit of Detection Matters: The Case for Benchmarking Severe Acute Respiratory Syndrome Coronavirus 2 Testing. Clin Infect Dis. 2021;73(9):e3042–e3046.

[20] Jacot D, Greub G, Jaton K, Opota O. Viral load of SARS-CoV-2 across patients and compared to other respiratory viruses. Microbes Infect. 2020;22(10):617–621.

[21] Jacot *et al.* only measure the initial sample of a given patient, but these patients were tested at different points during disease progression [20].

[22] Brandolini M, Taddei F, Marino MM, Grumiro L, Scalcione A, Turba ME, et al. Correlating qRT-PCR, dPCR and Viral Titration for the Identification and Quantification of SARS-CoV-2: A New Approach for Infection Management. Viruses. 2021;13(6):1022.

[23] Ke R, Zitzmann C, Ho DD, Ribeiro RM, Perelson AS. In vivo kinetics of SARS-CoV-2 infection and its relationship with a person's infectiousness. Proc Natl Acad Sci U S A. 2021;118(49):e2111477118.

[24] CDC. Duration of isolation and precautions for Adults with COVID-19; 2020. Accessed: 2020-11-17. `https://www.cdc.gov/coronavirus/2019-ncov/hcp/duration-isolation.html`.

[25] Wölfel R, Corman VM, Guggemos W, Seilmaier M, Zange S, Müller MA, et al. Virological assessment of hospitalized patients with COVID-2019. Nature. 2020;581(7809):465–469.

[26] Bullard J, Dust K, Funk D, Strong JE, Alexander D, Garnett L, et al. Predicting infectious SARS-CoV-2 from diagnostic samples. Clin Infect Dis. 2020;71(10):2663–2666.

[27] Lu J, Peng J, Xiong Q, Liu Z, Lin H, Tan X, et al. Clinical, immunological and virological characterization of COVID-19 patients that test re-positive for SARS-CoV-2 by RT-PCR. EBioMedicine. 2020;59:102960.

[28] Arons MM, Hatfield KM, Reddy SC, Kimball A, James A, Jacobs JR, et al. Presymptomatic SARS-CoV-2 infections and transmission in a skilled nursing facility. N Engl J Med. 2020;382(22):2081–2090.

[29] Criteria for releasing COVID-19 patients from isolation. Accessed: 2020-11-17. `https://www.who.int/news-room/commentaries/detail/criteria-for-releasing-covid-19-patients-from-isolation`.

[30] Sia SF, Yan LM, Chin AWH, Fung K, Choy KT, Wong AYL, et al. Pathogenesis and transmission of SARS-CoV-2 in golden hamsters. Nature. 2020;583(7818):834–838.

[31] Martins M, Fernandes MHV, Joshi LR, Diel DG. Age-related susceptibility of ferrets to SARS-CoV-2 infection. J Virol. 2021; p. JVI0145521.

[32] Goyal A, Cardozo-Ojeda EF, Schiffer JT. Potency and timing of antiviral therapy as determinants of duration of SARS-CoV-2 shedding and intensity of inflammatory response. Science Advances. 2020;6(47):eabc7112.

[33] Goyal A, Reeves DB, Cardozo-Ojeda EF, Schiffer JT, Mayer BT. Viral load and contact heterogeneity predict SARS-CoV-2 transmission and super-spreading events. Elife. 2021;10:e63537.

[34] Matteo Chiesa And Sergio. PCR positives: what do they mean? - The Centre for Evidence-Based Medicine; 2020. Accessed: 2020-11-17. https://www.cebm.net/covid-19/pcr-positives-what-do-they-mean/.

[35] Johnson GR, Morawska L, Ristovski ZD, Hargreaves M, Mengersen K, Chao CYH, et al. Modality of human expired aerosol size distributions. J Aerosol Sci. 2011;42(12):839–851.

[36] Bake B, Larsson P, Ljungkvist G, Ljungström E, Olin AC. Exhaled particles and small airways. Respir Res. 2019;20(1):8.

[37] Dhand R, Li J. Coughs and sneezes: their role in transmission of respiratory Viral Infections, Including SARS-CoV-2. Am J Respir Crit Care Med. 2020;202(5):651–659.

[38] Hernes SS, Quarsten H, Hagen E, Lyngroth AL, Pripp AH, Bjorvatn B, et al. Swabbing for respiratory viral infections in older patients: a comparison of rayon and nylon flocked swabs. Eur J Clin Microbiol Infect Dis. 2011;30(2):159–165.

[39] Hernes SS, Quarsten H, Hamre R, Hagen E, Bjorvatn B, Bakke PS. A comparison of nasopharyngeal and oropharyngeal swabbing for the detection of influenza virus by real-time PCR. Eur J Clin Microbiol Infect Dis. 2013;32(3):381–385.

[40] Wyllie AL, Fournier J, Casanovas-Massana A, Campbell M, Tokuyama M, Vijayakumar P, et al. Saliva or nasopharyngeal swab specimens for detection of SARS-CoV-2. N Engl J Med. 2020;383(13):1283–1286.

[41] Marty FM, Chen K, Verrill KA. How to obtain a nasopharyngeal swab specimen. N Engl J Med. 2020;382(22):e76.

[42] Wang H, Liu Q, Hu J, Zhou M, Yu MQ, Li KY, et al. Nasopharyngeal swabs are more sensitive than oropharyngeal Swabs for COVID-19 Diagnosis and Monitoring the SARS-CoV-2 Load. Front Med. 2020;7:334.

[43] Zasada AA, Zacharczuk K, Woźnica K, Główka M, Ziółkowski R, Malinowska E. The influence of a swab type on the results of point-of-care tests. AMB Express. 2020;10(1):46.

[44] Liu R, Yi S, Zhang J, Lv Z, Zhu C, Zhang Y. Viral load dynamics in sputum and nasopharyngeal swab in patients with COVID-19. J Dent Res. 2020;99(11):1239–1244.

[45] Stadnytskyi V, Bax CE, Bax A, Anfinrud P. The airborne lifetime of small speech droplets and their potential importance in SARS-CoV-2 transmission. Proc Natl Acad Sci U S A. 2020;117(22):11875–11877.

[46] Morawska L, Johnson GR, Ristovski ZD, Hargreaves M, Mengersen K, Corbett S, et al. Size distribution and sites of origin of droplets expelled from the human respiratory tract during expiratory activities. J Aerosol Sci. 2009;40(3):256–269.

[47] The factor of 17.7 is obtained bycomparing the average of the "aah-v-p" and "c-v-p" number concentrations with the average of the "b-n-m" and "b-n-n" concentrations. Note that in the vocalization experiments, equal time is spent talking and quiet, so we remove the "background" concentration due to the time spent only breathing. The factor of 45.7 is obtained the same way, summing the volume contributions of each measured diameter bucket from 0.8 μm to 5.5 μm.

[48] Asadi S, Wexler AS, Cappa CD, Barreda S, Bouvier NM, Ristenpart WD. Aerosol emission and superemission during human speech increase with voice loudness. Sci Rep. 2019;9(1):2348.

[49] Loudon RG, Roberts RM. Droplet expulsion from the respiratory tract. Am Rev Respir Dis. 1967;95(3):435–442.

[50] Evans MJ. Avoiding COVID-19: Aerosol Guidelines. medRxiv. 2020;(2020.05.21.20108894).

[51] Smith SH, Somsen GA, van Rijn C, Kooij S, van der Hoek L, Bem RA, et al. Aerosol persistence in relation to possible transmission of SARS-CoV-2. Phys Fluids. 2020;32(10):107108.

[52] Mikszewski A, Stabile L, Buonanno G, Morawska L. The airborne contagiousness of respiratory viruses: A comparative analysis and implications for mitigation. Geoscience Frontiers. 2021; p. 101285.

[53] Effros RM, Biller J, Foss B, Hoagland K, Dunning MB, Castillo D, et al. A simple method for estimating respiratory solute dilution in exhaled breath condensates. Am J Respir Crit Care Med. 2003;168(12):1500–1505.

[54] Effros RM, Dunning MB 3rd, Biller J, Shaker R. The promise and perils of exhaled breath condensates. Am J Physiol Lung Cell Mol Physiol. 2004;287(6):L1073–80.

[55] Effros RM, Casaburi R, Porszasz J, Morales EM, Rehan V. Exhaled breath condensates: analyzing the expiratory plume. Am J Respir Crit Care Med. 2012;185(8):803–804.

[56] Although the individual reduction factor measurements vary widely, the dilution estimates are much tighter, at 20,472 ± 2,516 for cation measurements, 21,019 ± 2,427 for conductivity measurements, and 18,818 ± 2,402 for urea measurements [53].

[57] Brandis K. Fluid Physiology (Chapter 3.2). Libretext; 2020. Available from: `https://batch.libretexts.org/print/Letter/Finished/med-11215/Full.pdf`.

[58] In a later paper, Effros et al. cite a dilution factor of approximately 10,000 [55].

[59] Chia PY, Coleman KK, Tan YK, Ong SWX, Gum M, Lau SK, et al. Detection of air and surface contamination by SARS-CoV-2 in hospital rooms of infected patients. Nat Commun. 2020;11(1):2800.

[60] Yan J, Grantham M, Pantelic J, Bueno de Mesquita PJ, Albert B, Liu F, et al. Infectious virus in exhaled breath of symptomatic seasonal influenza cases from a college community. Proc Natl Acad Sci U S A. 2018;115(5):1081–1086.

[61] Zhang N, Li Y. Transmission of Influenza A in a Student Office Based on Realistic Person-to-Person Contact and Surface Touch Behaviour. Int J Environ Res Public Health. 2018;15(8):1699.

[62] Huang SH, Kuo YM, Lin CW, Ke WR, Chen CC. Experimental characterization of aerosol suspension in a rotating drum. Aerosol Air Qual Res. 2019;19(4):688–697.
