# Supplementary material for: Finding the infectious dose for COVID-19 by applying an airborne-transmission model to superspreader events
Source: PLoS One. 2022 Jun 9;17(6):e0265816. doi: 10.1371/journal.pone.0265816 (PMC9182663; doi:10.1371/journal.pone.0265816)
Supplement: S2 Text — (PDF) [file pone.0265816.s003.pdf]

S2: Discussion of Direct SARS-CoV2 Exhalation Measurements

Ideally, this work could have used direct measurements of SARS-CoV-2 exhalation for the virion emission rate S, rather than inferring it as a product of viral density ρ and exhaled volume ϕ. However, direct measurements of S span several orders of magnitude. Using exhaled breath condensate, Ma *et al.* were able to detect virus in 14 out of 52 hospitalized patients, with a median emission rate (interquartile range) while breathing ($S_{\text{breathing}}$) of 7.5 $\times 10^5$ (2.1 $\times 10^5$ - 1.5 $\times 10^6$) copies/hr [1]. Malik *et al* also used exhaled breath condensate, finding detectable virus in 70 out of 100 patients but with a interquartile emission rate more than an order of magnitude smaller: (1.2 $\times 10^4$ - 1.0 $\times 10^5$ copies/hr). Finally, Coleman *et al*, using a different measurement technique, find $S_{\text{breathing}}$ 2-3 orders of magnitude lower (median 1.3 $\times 10^2$, interquartile range (0, 4.6 $\times 10^2$ copies/hr)). In this work, we use $S_{\text{breathing}}$ = 6.0 $\times 10^3$ copies/hr, which is $\sim$ 2-17 times smaller than the middle (Malik) measurement. Had we used this measurement, the estimated values of N_0 would be an order of magnitude larger. We are unable to reconcile these three measurements. However, as we discuss below, the difference between the two exhaled breath measurements may be explainable by an unusual conversion of Ct value to viral copies used in Ma *et al*.

Discussion of Ma *et al.* Exhaled Breath Measurement

Ma *et al.* used EBC to measure SARS-Cov-2 emissions in breath and found positive readings in 14 out of 52 (26%) of the EBC samples. They inferred an emission rate of 1.03 $\times$ 10^5 - 2.25 $\times$ 10^7 virus copies/hour [1], which is substantially higher than the $\sim$6,000 copies/hour implied by our breathing calculation. Here, we discuss this measurement, with a particular focus on the conversion of Ct values to copies/mL.

Ma *et al.* collected EBC samples from hospitalized COVID patients. The RNA from these samples was extracted into 70 μL samples and quantified using RT-PCR. The limit of detection was assumed to be 100 copies/μL, the amplification efficiency 75%, and the maximum cycle threshold 39.5, so that the copies/μL = 100 $\times$ $1.75^{(39.5 - \text{Ct})}$. Since the EBC samples were collected for 5 minutes, the emission rate (virions/hr) was estimated as $S_{\text{breathing}}$ = 60/5 $\times$ 70 $\times$ 100 $\times$ $1.75^{(39.5 - \text{Ct})}$. The maximal and minimal Ct values in the sample were 39.14 and 29.51, respectively; therefore, these values correspond to emission rates of 1.03 $\times$ 10^5 - 2.25 $\times$ 10^7 virions/hour, respectively, in agreement with the range reported [1]. The reported average Ct value of 35.54 corresponds to an emission rate of 7.70 $\times$ 10^5 copies/hour.

These emission rates, as noted in the main text, are far above the 6 $\times$ 10^3 copies/mL used for breathing in the calculations here. While differences in viral loads between presymptomatic patients and hospitalized

patients could account for some of the gap, we note that the emission rates found in Ma *et al.* imply exceptionally high viral loads in breathed aerosols. Ma collected 200 μL of condensed breath during a 5 minute collection period (including a 10 μL scavenging liquid.) If we assume a dilution factor of 20000 for respiratory fluid to EBC collected [2, 3], then if 200 μL of EBC are collected in 5 minutes, respiratory fluid would be released at a rate of 1.2 $\times10^{-4}$ mL/hour, similar to our assumed value of 6.0 $\times10^{-4}$ mL/hour. The resultant range of viral loads in aerosolized particles in the reported range of emission rates (1.03 $\times$ 10^5 - 2.25 $\times$ 10^7 virions/hour) is 8.56 $\times$ 10^8 - 1.88 $\times10^{11}$ virions/mL. While such a range is in principle possible, it is far above the ranges observed in other studies. For instance, in hospitalized patients, Wölfel *et al.* observed an average viral load in sputum of 7.00 $\times10^6$ copies/mL and a maximum viral load of 2.35 $\times10^9$ virions/mL [4]; Pan *et al.* found a median viral load in sputum for hospitalized patients of 7.52 $\times10^5$ copies/mL, and a maximum load of 1.34 $\times10^{11}$, obtained from a patient who died from the disease [5].

We believe that the differences between our rate of emission and that of Ma *et al.* may be due to the method used to convert Ct values to viral load. Whereas Ma *et al.* assumed a limit of detection of 100 copies/μL, other studies find a limit of detection (obtained by calibration) which is approximately 1000 times lower and of order 100 copies/mL [6, 7]. As such, for a given Ct value, these other studies will find a much lower viral load (2-3 orders of magnitude, depending on the efficiency of PCR replication and limit of detection). Ma *et al.* convert Ct values to copies/mL according to Log_{10} (copies/mL) = 14.60 - 0.24 Ct. For the average Ct value of 35.54 reported by Ma, this conversion implies a viral load in the EBC sample (taking into account a dilution factor of 70/200 for the 200 μL EBC sample vs. 70 μL extracted DNA sample) of $\sim$5.5 Log_{10} copies/mL. In contrast, for the same Ct value, Zou (Log_{10} (copies/mL = 14.11 - 0.32 Ct), Jones (Log_{10} (copies/mL) = 14.16 - 0.30 Ct), and Jacot (Log_{10} (copies/mL) = 13.04 - 0.27Ct) would find 2.2, 3.1, 3.0 Log_{10} copies/mL, respectively. If we repeat the same calculation for the virions/hour, using the average of the viral loads implied by the Zou, Jones, and Jacot Ct conversions, we arrive at a range of 1.8 $\times10^2$ - 1.1 $\times$ 10^5 copies/hour - a wide interval, but one which includes our emission rate of 6.0 $\times10^3$ copies/hour. Our intent here is not to achieve quantitative agreement, but rather, to point out that the conclusion that breathing emits 10^5 - 10^7 copies/hour is dependent on the conversion of Ct to viral load, which in turn is dependent on an unusually high assumed limit of detection.

References

[1] Ma J, Qi X, Chen H, Li X, Zhang Z, Wang H, et al. COVID-19 patients in earlier stages exhaled millions of SARS-CoV-2 per hour. Clin Infect Dis. 2021;72(10):e652–e654.

[2] Effros RM, Biller J, Foss B, Hoagland K, Dunning MB, Castillo D, et al. A simple method for estimating respiratory solute dilution in exhaled breath condensates. Am J Respir Crit Care Med. 2003;168(12):1500–1505.

[3] Effros RM, Dunning MB 3rd, Biller J, Shaker R. The promise and perils of exhaled breath condensates. Am J Physiol Lung Cell Mol Physiol. 2004;287(6):L1073–80.

[4] Wölfel R, Corman VM, Guggemos W, Seilmaier M, Zange S, Müller MA, et al. Virological assessment of hospitalized patients with COVID-2019. Nature. 2020;581(7809):465–469.

[5] Pan Y, Zhang D, Yang P, Poon LLM, Wang Q. Viral load of SARS-CoV-2 in clinical samples. Lancet Infect Dis. 2020;20(4):411–412.

[6] Kleiboeker S, Cowden S, Grantham J, Nutt J, Tyler A, Berg A, et al. SARS-CoV-2 viral load assessment in respiratory samples. J Clin Virol. 2020;129:104439.

[7] Arnaout R, Lee RA, Lee GR, Callahan C, Cheng A, Yen CF, et al. The Limit of Detection Matters: The Case for Benchmarking Severe Acute Respiratory Syndrome Coronavirus 2 Testing. Clin Infect Dis. 2021;73(9):e3042–e3046.
