# Supplementary material for: Finding the infectious dose for COVID-19 by applying an airborne-transmission model to superspreader events
Source: PLoS One. 2022 Jun 9;17(6):e0265816. doi: 10.1371/journal.pone.0265816 (PMC9182663; doi:10.1371/journal.pone.0265816)
Supplement: S3 Text — (PDF) [file pone.0265816.s004.pdf]

# S3: Risks of Everyday Activities

## Global Assumptions

In analyzing the risks of everyday situations, in addition to the usual input parameters of breathing rate, volume, and so on, we make a few global assumptions:

- Source strength: we use the averages from the case studies of 10 quanta/h for breathing and 460 quanta/h for talking.
- Mask effect: For mask efficacy, we assume that masks block 71% of outgoing viral particles [1] and 50% of incoming particles, so that masks worn by both infected and susceptible individuals reduce the overall inhaled viral particles by ~85%.
- Filtration effect: assume that we have a filter that exchanges the air at a rate $R$ (i.e. passes the air in the room through the filter at $R$ times per hour), and that each pass through the filter reduces the amount of the virus by a factor $Q < 1$. For example, if a filter removes 90% of virions on each pass, $Q = 0.1$; a HEPA filter, which removes 99.97% of particles, has $Q = 0.0003$. In the limit that the filter airflow is weak and the room remains well-mixed at all times, the filter is equivalent to adding an amount $R$ (1 - $Q$) to the air exchange rate and $\lambda_3 = R\ (1 - Q)$. However, if the filter airflow is sufficiently powerful that the filtration time is small compared to the mixing time, then after time $t$, the number of times through the filter is $R\ t$ and the viral reduction factor is $Q^{R\ t} = \exp(R \ln(Q)\ t) = \exp(-\lambda_3\ t)$ -- i.e. $\lambda_3 = -R \ln(Q)$ [2].

## Representative Values of Air Exchange Rate $\lambda_{air}$

The air exchange rate $\lambda_{air}$varies depending on the setting.

**Residential homes** can be quite "tight", meaning that air may turn over less than one time per hour: the EPA recommends using $\lambda_{air}$ = 0.45/h (i.e. the air turns over every ~1/0.45 ~ 2 hours) for residential homes broadly [3], while the California Department of Public Health recommends using $\lambda_{air}$ = 0.23/hr based on measurements in newer homes, which tend to have better insulation and less air exchange with the outside [4].

**Office buildings and institutional buildings** usually have higher air exchange rates – circa 1.0/h in schools [5], dormitories, etc; the EPA recommends a mean value of 1.5/h for nonresidential buildings [3].

**Motor vehicles** typically have high air exchange rates. A relatively modern car with the windows closed and vent off was measured to have an ACH of ∼1.8/h when stationary, 5.6/h at 35 mph, and 13.5 at 55 mph. With higher outside ventilation, these figures climb to 10.7/h, 35.7/h, and 55/h [6,7]. Measurements when vehicles are traveling with air in "recirculate" mode find that when vehicles are traveling 60 km/h (∼37 mph), ACH range from ∼2.5 to ∼5.5 [8]. Note, however, that in tour buses, particularly those with air set to recirculation mode, the air exchange rates have been characterized as "severely lacking," so that buses may have significantly lower ACH if the windows are closed and the vents are closed [9].

**Airplanes** have 20-30 air exchanges per hour, about half of which is recirculated air, and half of which is outside air. Note that the recirculated air generally will pass through a strong filter (e.g. a HEPA filter, which eliminates ∼99+% of viruses per pass (i.e. is very similar to fresh air) [10]. American Airlines, for instance, claims that the cabin air recirculates every 2-4 minutes through a HEPA filter which eliminates 99.97% of particles, including viral particles [11]. Recent measurements of dispersed aerosols on widebody Boeing aircraft are in agreement with this air change rate, finding 32-35 air changes per hour [12].

## Calculated Risks

The details of each scenario are shown in the Supporting Information Spreadsheet S5 (tab "Scenario Calculator") in the Supporting Information, and the summary risks are shown in Table 1. In cases with small numbers of people (e.g. office, classroom), we assume a single index patient; in cases with large numbers of people (grocery, airplane), we assume a number of index patients dependent on an infection rate and the venue capacity. In each case,we present the probability of infection assuming the index patient(s) spends some time breathing and some time talking-- for instance, in an office, talking 15% of the time. We make several comments about the calculated risks:

- The risk figures here apply only to the original variant and are necessarily imprecise because quanta emission rates are not known with certainty and may vary from person to person. The infection probabilities for many of the scenarios (dinner party, office, classroom, taxi) are conservative because they assume the presence of an infected individual; the absolute risks need to be multiplied by the infection prevalence rate. Furthermore, the quanta underlying the probabilities correspond to times at or close to peak infectivity, within 1 day of peak viral load; the risks will likely be much lower earlier and later. On the other hand, our calculations only take into account the risks of aerosol transmission and attribute no risk to larger droplets or fomites, which may be significant in cases with close contact.

Our calculations also neglect infection via aerosol exposure to the eyes, which may also play a role.

- Nonetheless, the broad trends across the various scenarios emphasize the importance of the key variables for aerosol transmission: the amount of talking versus breathing, the presence or absence of masks, the number of air changes per hour, the interaction time, and the volume through which the aerosols spread.

- Household interactions, as modeled by the "Dinner Party" scenario, are particularly risky for aerosol transmission, with an estimated 2/3 probability of an infected person passing on the disease. Air exchange rates tend to be low ($\sim$1x/hr or less) in most residential settings, and home dwellers are both unmasked and speak frequently. Even a one-hour dinner among a family with one infected individual would have a 22% risk of transmission under the same assumption. Considering the additional risk of droplet transmission, it is unsurprising that household attack rates of greater than 50% have been observed [13].

- The volume of the space in question matters. Very large volumes, such as grocery stores, pose little risk for a visitor (but more substantial integrated risk to an employee over many days)–despite the statistical likelihood of one or more infected persons present in the store (we note, however, that the well-mixed approximation is unlikely to be strictly correct in this case). Conversely, a taxi ride is typically short but presents an extremely small volume ($\sim$3 m $^3$), so if not well ventilated, the risks are intermediate, at 5%, even if all parties are masked. Unmasked sharing increases automobile risks dramatically (including droplet risk, which is not modeled here), as emphasized by a recent case of six unmasked UK National Health Service nurses becoming infected through ride sharing [14].

- Very strong air filtration can offset small, dense conditions, as in the case for commercial aircraft, where we assume 25 air changes per hour and calculate a <1% risk of infection for a 5 hour flight from aerosols, assuming passengers are masked. This low risk of aerosol infection is in line with other recent studies [12, 15]. We emphasize, however, that this calculation also likely underestimates the risk of infection: not only does it assume perfect mask compliance and neglect the effect of close-range droplet transmission/fomites, but also, aircraft have strong airflows so that the well-mixed assumption is not met. Studies of airplane transmission show a strong spatial dependence in attack rate [16–18]; the effective volume that is well-mixed is smaller than the assumed total cabin volume [19].

- The relative risk of a school classroom versus an indoor dining venue presents an interesting comparison. Indoor dining presents a larger volume and slightly higher air exchange rate than a household dinner party, but due to the unmasked interactions and likelihood of talking, we find a ∼9% risk of transmission for a visitor (an employee is at much higher risk), assuming the presence of a single infected person (which is likely for a restaurant with a daily throughput of 100 or more patrons). As discussed above, a classroom can be either quite unsafe (27% chance of transmission) or quite safe (1% chance of transmission), depending on masking and air exchange; in addition, unlike restaurants, schools have a greater ability to prevent potentially infected students from entering, through e.g. daily health attestations. Finally, we note that we assume that the class time is unusually long (6 hours), as some schools are attempting to create isolated pods of students.

Table 1: **Summary Risks by Scenario. Each scenario assumes a certain percentage of time breathing versus talking, as well as the use, or not, of masks and filters. See the spreadsheet S7 (tab "scenario Calculator") in Supporting Information for the detailed assumptions of each scenario; these assumptions can be changed by the interested reader.**

| | Assumptions | | | | p(infection) |
|---|---|---|---|---|---|
| Case | # infected present | Masks | % time talking | Filter Efficiency | Scenario |
| Dinner Party | 1 | No | 25% | (no filter) | 66.8% |
| Bad Office | 1 | No | 15% | (no filter) | 24.1% |
| Good Office | 1 | Yes | 15% | 90% | 0.8% |
| Bad Classroom | 1 | No | 5% | (no filter) | 27.3% |
| Good Classroom | 1 | Yes | 5% | 80% | 1.2% |
| Taxi | 1 | Yes | 5% | (no filter) | 4.9% |
| Grocery Shopping [20] | 2 | Yes | 5% | (no filter) | 0.01% |
| Commercial Flight | 2 | Yes | 15% | 99% | 0.4% |
| Indoor Dining | 1 | No | 33% | (no filter) | 8.8% |

# References


[1] Milton DK, Fabian MP, Cowling BJ, Grantham ML, McDevitt JJ. Influenza virus aerosols in human exhaled breath: particle size, culturability, and effect of surgical masks. PLoS Pathog. 2013;9(3):e1003205.

[2] Augenbraun BL, Lasner ZD, Mitra D, Prabhu S, Raval S, Sawaoka H, et al. Assessment and mitigation of aerosol airborne SARS-CoV-2 transmission in laboratory and office environments. J Occup Environ Hyg. 2020;17(10):447–456.

[3] U S EPA Office of Research and Development. Chapter 19 (Building Characteristics). In: Exposure Factors Handbook. Washington DC 20460: U.S. Environmental Protection Agency; 2018.

[4] Chen W, Persily AK, Hodgson AT, Offermann FJ, Poppendieck D, Kumagai K. Area-specific airflow rates for evaluating the impacts of VOC emissions in U.S. single-family homes. Build Environ. 2014;71:204–211.

[5] You Y, Niu C, Zhou J, Liu Y, Bai Z, Zhang J, et al. Measurement of air exchange rates in different indoor environments using continuous CO2 sensors. J Environ Sci. 2012;24(4):657–664.

[6] Ott W, Klepeis N, Switzer P. Air change rates of motor vehicles and in-vehicle pollutant concentrations from secondhand smoke. J Expo Sci Environ Epidemiol. 2008;18(3):312–325.

[7] Rodes C, Sheldon L, Whitaker D, Clayton A, Fitzgerald K, Flanagan J, et al. Measuring concentrations of selected air pollutants inside California vehicles. California Environmental Protection Agency; 1998. Contract 95-339.

[8] Tong Z, Li Y, Westerdahl D, Adamkiewicz G, Spengler JD. Exploring the effects of ventilation practices in mitigating in-vehicle exposure to traffic-related air pollutants in China. Environ Int. 2019;127:773–784.

[9] Chiu CF, Chen MH, Chang FH. Carbon Dioxide Concentrations and Temperatures within Tour Buses under Real-Time Traffic Conditions. PLoS One. 2015;10(4):e0125117.

[10] Hunt EH, Space DR. The Airplane Cabin Environment: Issues Pertaining to Flight Attendant. In: Comfort, International In-flight Service Management Organization Conference; 1994.

[11] American Airlines. How HEPA Filters Have Been Purifying Cabin Air Since the 1990s; 2020. Accessed: 2020-9-NA. https://news.aa.com/news/news-details/2020/

How-HEPA-Filters-Have-Been-Purifying-Cabin-Air-Since-the-1990s-FLT-06/default.aspx#:~:text=The%20HEPA%20filters%20in%20use,engine%20compressor%20on%20the%20outside.

[12] Silcott D, Kinahan S, Santarpia J, Silcott B, Silcott P, Silcott B, et al. TRANSCOM/AMC commercial aircraft cabin aerosol dispersion tests. National Strategic Research Institute Lincoln United States; 2020.

[13] Grijalva CG. Transmission of SARS-COV-2 Infections in Households — Tennessee and Wisconsin, April–September 2020. MMWR Morb Mortal Wkly Rep. 2020;69.

[14] Campbell D. Six NHS staff contract Covid after car sharing without wearing masks. The Guardian. 2020.

[15] Faculty, of Harvard T H Chan School of Public Health S. Assessment of Risks of SARS-CoV-2 Transmission During Air Travel and Non-Pharmaceutical Interventions to Reduce Risk. Harvard T.H. Chan School of Public Health; 2020. Available from: https://cdn1.sph.harvard.edu/wp-content/uploads/sites/2443/2020/10/HSPH-APHI-Phase-One-Report.pdf.

[16] Khanh NC, Thai PQ, Quach HL, Thi NAH, Dinh PC, Duong TN, et al. Transmission of SARS-CoV 2 during long-haul flight. Emerg Infect Dis. 2020;26(11):2617–2624.

[17] Swadi T, Geoghegan JL, Devine T, McElnay C, Sherwood J, Shoemack P, et al. Genomic Evidence of In-Flight Transmission of SARS-CoV-2 Despite Predeparture Testing. Emerg Infect Dis. 2021;27(3):687–693.

[18] Speake H, Phillips A, Chong T, Sikazwe C, Levy A, Lang J, et al. Flight-associated transmission of severe acute respiratory syndrome coronavirus 2 corroborated by whole-genome sequencing. Emerg Infect Dis. 2020;26(12):2872–2880.

[19] In the Transcom and Harvard studies, the authors calculate the times to be exposed to 1 quanta, which are of order 50 or more hours; we use these values to imply a quanta per hour exposure, which we compare to our values for a 5 hour flight. We also note that it is somewhat odd to express the risks as the time to reach a certain arbitrary quanta exposure, since the risks smoothly increase as the number of exhaled quanta increase; if it takes 50 hours to get to 1 quanta on a flight, then after 10 hours, the risk of infection will be 18%, which is sufficient to induce a superspreading event.

[20] The well-mixed approximation is likely not strictly correct in all cases. For example, the volume of the supermarket is so large that the timescales do not justify the well-mixed approximation over the entire volume; however, even if one used a much smaller volume, the risks would still be very low. Similarly, in the case of an airplane, there is active airflow and recirculation which may create somewhat distinct, smaller zones similar to those observed in the Guangzhou restaurant case.
