# Supplementary material for: Finding the infectious dose for COVID-19 by applying an airborne-transmission model to superspreader events
Source: PLoS One. 2022 Jun 9;17(6):e0265816. doi: 10.1371/journal.pone.0265816 (PMC9182663; doi:10.1371/journal.pone.0265816)
Supplement: S4 Text — (PDF) [file pone.0265816.s005.pdf]

S4: Guangzhou Restaurant

In the spreadsheet S5 in the Supporting Information (tab "Dose calculation"), we also consider a well-known case of a restaurant in Guangzhou, where active air conditioning appeared to spread the virus from an index patient to nearby tables in the recirculation zone [1]. One strength of this case is that the airflow characteristics are well-known and well-modeled. Li *et al.* have analyzed this case in detail as a probable case of aerosol transmission, including performing tracer gas measurements to find an average air exchange rate of $\sim$0.67 h^{-1} and performing detailed computational fluid dynamics simulations [2]. However, the attack rate is not well defined: 3 out of 4 people in one family, and 2 out of 7 people in another family (both families in the recirculation zone) were infected, but it is unknown how many of these infections were due to exposure at the restaurant. Lu *et al.* note that it is likely that all people were infected at the restaurant, but that it is also possible that family transmission played a role, so the attack rate ranges from 2/11 to 5/11 [1]. In addition, it is unclear what volume to apply in a simple well-mixed model. The air conditioned zone is not well-mixed with the other spaces in the restaurant and carried a higher concentration of aerosols than other locations, but other areas in the restaurant had similar concentrations. Li *et al.* calculated and measured (in their table S1) that the air conditioned zone where the infections took place had a tracer gas density 2-3 times that in more remote regions of the restaurant, where no patrons were infected [2]. Taking all of these uncertainties into account, we can still perform a stylized calculation assuming that the relevant volume is either the zone of the air conditioning or the entire restaurant. We find a similar range of N_0 = 499-948 if we assume all infected individuals were infected in the restaurant, and a range of N_0 = 1,507-2,415 if only one member of each family was infected at the restaurant.

References

[1] Lu J, Gu J, Li K, Xu C, Su W, Lai Z, et al. COVID-19 Outbreak Associated with Air Conditioning in Restaurant, Guangzhou, China, 2020. Emerg Infect Dis. 2020;26(7):1628–1631.

[2] Li Y, Qian H, Hang J, Chen X, Cheng P, Ling H, et al. Probable airborne transmission of SARS-CoV-2 in a poorly ventilated restaurant. Build Environ. 2021;196:107788.
